# Combination of WFDC2, CHI3L1, and KRT19 in Plasma Defines a Clinically Useful Molecular Phenotype Associated with Prognosis in Critically Ill COVID-19 Patients

**DOI:** 10.1007/s10875-022-01386-3

**Published:** 2022-11-04

**Authors:** Takeshi Ebihara, Tsunehiro Matsubara, Yuki Togami, Hisatake Matsumoto, Jotaro Tachino, Hiroshi Matsuura, Takashi Kojima, Fuminori Sugihara, Shigeto Seno, Daisuke Okuzaki, Haruhiko Hirata, Hiroshi Ogura

**Affiliations:** 1 Department of Traumatology and Acute Critical Medicine, Osaka University Graduate School of Medicine, Suita, Osaka Japan; 2 Osaka Prefectural Nakakawachi Emergency and Critical Care Center, Higashiosaka, Osaka Japan; 3 Laboratory for Clinical Investigation, Osaka University Hospital, Suita, Osaka Japan; 4 Core Instrumentation Facility, Immunology Frontier Research Center and Research Institute for Microbial Diseases, Osaka University, Osaka, Japan; 5 Department of Bioinformatic Engineering, Graduate School of Information Science and Technology, Osaka University, Osaka, Japan; 6 Genome Information Research Center, Research Institute for Microbial Diseases, Osaka University, Osaka, Japan; 7 Department of Respiratory Medicine and Clinical Immunology, Osaka University Graduate School of Medicine, Suita, Osaka Japan

**Keywords:** Biomarker, Cluster analysis, Network analysis, Plasma proteomics

## Abstract

**Background:**

COVID-19 is now a common disease, but its pathogenesis remains unknown. Blood circulating proteins reflect host defenses against COVID-19. We investigated whether evaluation of longitudinal blood proteomics for COVID-19 and merging with clinical information would allow elucidation of its pathogenesis and develop a useful clinical phenotype.

**Methods:**

To achieve the first goal (determining key proteins), we derived plasma proteins related to disease severity by using a first discovery cohort. We then assessed the association of the derived proteins with clinical outcome in a second discovery cohort. Finally, the candidates were validated by enzyme-linked immunosorbent assay in a validation cohort to determine key proteins. For the second goal (understanding the associations of the clinical phenotypes with 28-day mortality and clinical outcome), we assessed the associations between clinical phenotypes derived by latent cluster analysis with the key proteins and 28-day mortality and clinical outcome.

**Results:**

We identified four key proteins (WFDC2, GDF15, CHI3L1, and KRT19) involved in critical pathogenesis from the three different cohorts. These key proteins were related to the function of cell adhesion and not immune response. Considering the multicollinearity, three clinical phenotypes based on WFDC2, CHI3L1, and KRT19 were identified that were associated with mortality and clinical outcome.

**Conclusion:**

The use of these easily measured key proteins offered new insight into the pathogenesis of COVID-19 and could be useful in a potential clinical application.

**Supplementary Information:**

The online version contains supplementary material available at 10.1007/s10875-022-01386-3.

## Background

Although the majority of individuals infected by COVID-19 exhibit no or mild-to-moderate symptoms, approximately 5–20% of subjects hospitalized required prolong treatment in an intensive care unit (ICU) with invasive mechanical ventilation (IMV) [1–4]. In these cases, excessive inflammation following a COVID-19 infection might lead to systemic inflammatory response syndrome and multiple organ failure [5].

From a clinical perspective, it is important to evaluate the blood circulating proteins that can reflect the systemic inflammation. The evaluation process is easy and rapid, and most of the biomarkers used in the ICU are based on the circulating proteins [6]. Previously, we showed that a key network of cytokine proteins based on a blood circulating cytokine profile and combined key cytokines score was related to prognosis and severity in critically ill patients including those with sepsis and burn [7–9]. Therefore, we hypothesized that a key protein network would also play an important role in critical COVID-19.

Recently, researchers have investigated new therapeutic targets by combining unsupervised clustering analysis and key biological indicators in various diseases to clarify potential sub-phenotypes [10–12]. Using key proteins to identify COVID-19 patient sub-phenotypes with poor outcomes may enable the discovery of new therapeutic strategies and target populations.

The present study approach involved several datasets and a statistical approach. To obtain globally versatile results, we used two discovery cohorts (i.e., American and Japanese cohorts) that had different patient characteristics (e.g., age, sex, and race). To achieve the primary goal, we derived plasma proteins related to COVID-19 pathogenesis from an innovative method, Olink proteomics, by using the two different cohorts. The candidates were validated by a classical method, enzyme-linked immunosorbent assay (ELISA), in a validation cohort to determine key proteins. To achieve the secondary goal, we derived the clinical phenotypes based on these key proteins and assessed their associations with mortality and clinical outcome.

## Methods

### Cohort Data and Measurement of Plasma Proteins

We used data from three observational cohorts. The first discovery cohort comprised publicly available data provided by the Massachusetts General Hospital Emergency Department COVID-19 Cohort [13] with Olink Proteomics (Olink® Explore 1536; https://www.olink.com/mgh-covid-study/) (Supplemental Methods, Statistical analysis, and Results), which was conducted from March 2020 to April 2020. In this study, we used proteomics data of days 1 and 8.

The second discovery cohort was composed of COVID-19 patients who were admitted to Osaka University Hospital from July 2020 to February 2021. Blood samples were obtained from the patients on days 1 (day of admission) or 2 and days 6–8 and once from healthy volunteers who were enrolled via public poster advertisements. Plasma proteomics were performed by using Olink® Explore 1536.

The validation cohort was composed of COVID-19 patients admitted to Osaka University Hospital or Osaka Prefectural Nakakawachi Emergency and Critical Care Center from December 2020 to January 2021 and April 2021, who were treated with IMV. Blood samples were obtained from the patients until hospital discharge or death on day 1 (day of admission) and days 6–8 and once from healthy volunteers who were enrolled via public poster advertisements. The plasma proteins were measured by ELISA. Details of the discovery and validation cohorts are shown in the Supplemental Methods, Statistical analysis, and Results.

### Definition of Disease Severity: Critical and Non-critical

Acuity scores were based on the World Health Organization ordinal outcomes scale [14]: A1, dead; A2, intubated, survived; A3, hospitalized with oxygen; A4, hospitalized without oxygen; A5, discharged. Disease severity was classified according to the maximum acuity score (acuity max 1 or 2). We defined “critical” as acuity max 1 and 2 subjects and “non-critical” as acuity max 3, 4, and 5 subjects.

### Definition of Timing of Sample Collection: Phase 1 and Phase 2

For easy clinical application, day 1 referred to the day of visiting the emergency department or of admission to the hospitals in this study, not to the day of disease onset or testing as PCR positive. We defined two different types of measurement timing: phase 1, days 1–2, and phase 2, days 6–10.

### Definitions of Clinical Outcome: Early Recovery and Late Recovery

We defined the clinical outcome of patients who were treated with IMV for ≤ 12 days or not treated with IMV as early recovery and IMV > 12 days or 28-day non-survivors as late recovery as in our previous study [15]. We divided the COVID-19 patients of the second derivation cohort and the validation cohort into two groups based on early recovery and late recovery and assessed them.

### Statistical Analysis

In the first discovery cohort, patients were divided into the critical group and non-critical group, and differences in the expression of 1463 plasma proteins were evaluated. Instead of normalization to the total protein concentration, Olink proteomics data was normalized using three internal and three external controls that were used for quality control and data normalization. The proteins levels were expressed as values of normalized protein expression (NPX), which was an arbitrary unit on a log2 scale [16]. The difference in NPX was used to detect the difference of protein expressions as previously described [17]. Differential expression analysis was conducted using the Welch 2-sample *t*-test. The false discovery rate was calculated by the Benjamin-Hochberg method [18]. Proteins with false discovery rate < 0.01 and |NPX difference|> 1 were considered to be significantly expressed. The plasma proteins reaching significance in both phase 1 and phase 2 were extracted as candidates of the first discovery cohort. The phase 1 NPX values of the candidates were compared among acuity scores with the Kruskal–Wallis test.

In the second discovery cohort, the candidates from the first discovery cohort were evaluated. The patients were divided into those with early recovery and late recovery. The Dunnet test was used to evaluate the levels of each candidate of the first discovery cohort between the healthy volunteers and COVID-19 patients in each phase. The Welch 2-sample *t*-test was used to evaluate the differences in the levels of candidates of the first discovery cohort between the two groups in each phase. The correlation analysis between the number of days since onset and the candidates of the first discovery cohort was performed by Spearman’s rank correlation. The trends of the two groups (early recovery and late recovery) are shown by linear regressions (solid lines) with 95% confidence intervals (gray areas).

We also evaluated the protein co-expression network only for critical COVID-19 patients in the second discovery cohort. Protein co-expression network analysis was performed with the R package “WGCNA” (weighted gene co-expression network analysis) as previously described [19] using the day 1 data of patients with acuity max scores 1 and 2. The module network is displayed graphically using Cytoscape® software (www.cytoscape.org) version 3.8.0 [20]. The biological functions of the proteins in each module were investigated by performing GO (Gene Ontology) pathway analysis [21] and KEGG (Kyoto Encyclopedia of Gene and Genomes) pathway analysis [22]. The details of WGCNA are shown in the Supplemental Methods, Statistical analysis, and Results.

In the validation cohort, the candidates from the second discovery cohort were validated. The levels of the candidates were transformed to common logarithmic values to normalize data distributions before analysis. The Dunnet test was used to evaluate the levels of each candidate between the healthy volunteers and COVID-19 patients in each phase. Then, the patients were divided into two groups, those with early recovery and late recovery. The candidates of the second discovery cohort were compared by Wilcoxon rank sum test between the two groups in each phase. The plasma proteins that were significantly increased (*P* values < 0.05) in the patients with late recovery compared with those in the patients with early recovery in both phases were extracted as key proteins. The association of these key proteins with 28-day and hospital mortality was also evaluated. The levels of key proteins in both phases were compared between the 28-day survivors and 28-day non-survivors or between hospital survivors and non-survivors by Wilcoxon rank sum test. The associations between phase 1 key proteins and body mass index (BMI), age, and comorbidities were also analyzed by Wilcoxon rank sum test or correlation analysis using Spearman’s rank correlation. We also evaluated these key proteins in the patients who were not treated with IMV. Details of the characteristics of these patients and the method are described in the Supplemental Methods.

Latent class analysis (LCA) was performed using a combination of key proteins to identify the new clinical phenotypes. Phase 1 key proteins were transformed to common logarithmic values and scaled to become candidate variables for LCA. Because the high correlation of variables in LCA caused lower accuracy of model fit statistics with an overestimation of the true number of classes, the correlation matrix of the four key proteins was evaluated. One in any pair in which a strong correlation (Pearson correlation coefficient > 0.6) was observed was eliminated [23]. The optimal number of phenotypes was identified by evaluating the Bayesian information criterion (BIC), the appropriate size of each phenotype, and the misclassification rate of each phenotype [24, 25]. The optimal number of phenotypes was selected based on the largest BIC, considering the misclassification rate and interpretability [11]. The latent class analysis calculation was performed using the VarSelLCM package in R, in which the largest BIC is interpreted as optimal. Cumulative mortality is illustrated using Kaplan–Meier curves, and the phenotypes were compared by the log rank test.

A two-sided *P* < 0.05 was considered statistically significant. For all statistical analyses, a fully scripted data management pathway was created within the R environment for statistical computing, version 4.0.2 (R Foundation for Statistical Computing, Vienna, Austria). Categorical variables are reported as number and percentages, and significance was detected by χ^2^ or Fisher’s exact test. The continuous variables are described using mean and standard error or compared using the Mann–Whitney *U* test or Kruskal–Wallis rank sum test described using median and interquartile range (IQR) values. There were no missing data on plasma proteomics in the first and second discovery cohorts or on plasma proteins levels in the validation cohort. However, 5 of 113 patients (4%) in the validation cohort had missing values for BMI, but no imputation was made for this missing data.

## Results

**Fig. 1:**
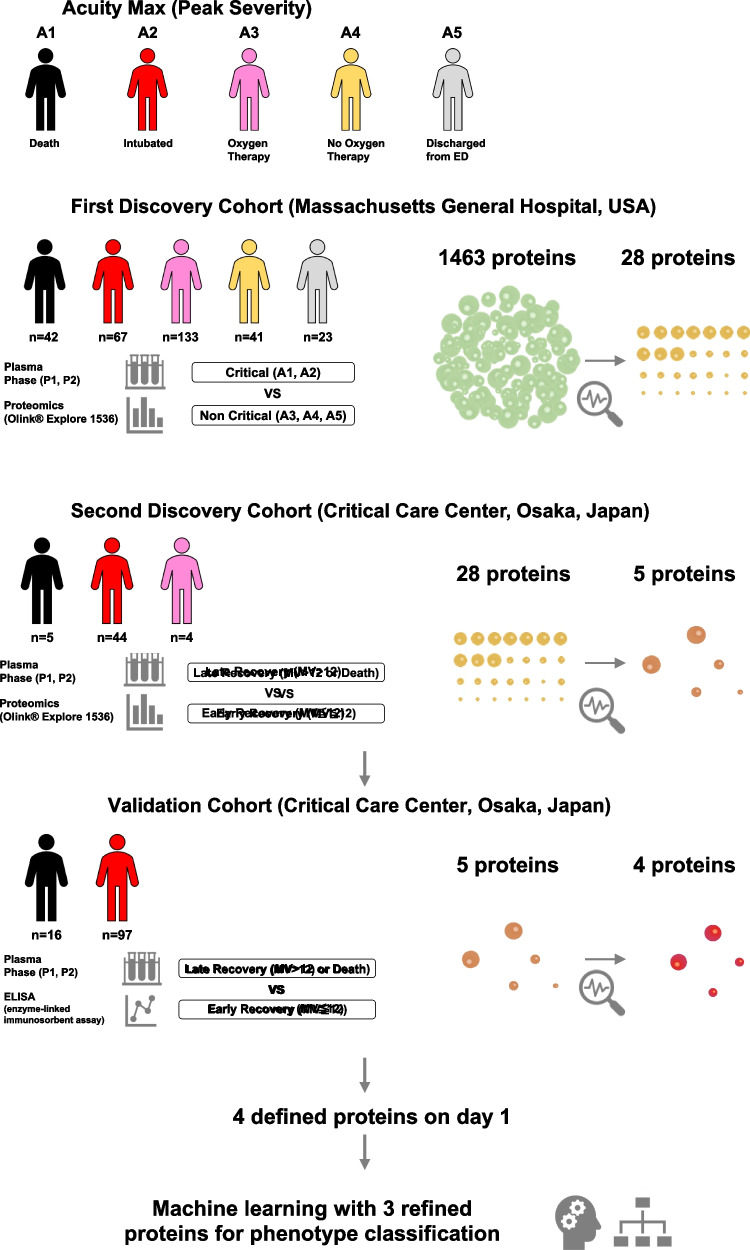
Flow chart of participants. COVID-19 phenotypes were evaluated using three plasma proteins including WFDC2, CHI3L1, and KRT19. P1: phase 1 (days 1–2), P2: phase 2 (days 6–10)

The study approach involved several datasets and the statistical approach shown in Fig. 1.

### Exploration of Candidate Plasma Proteins from First and Second Discovery Cohorts

**Fig. 2:**
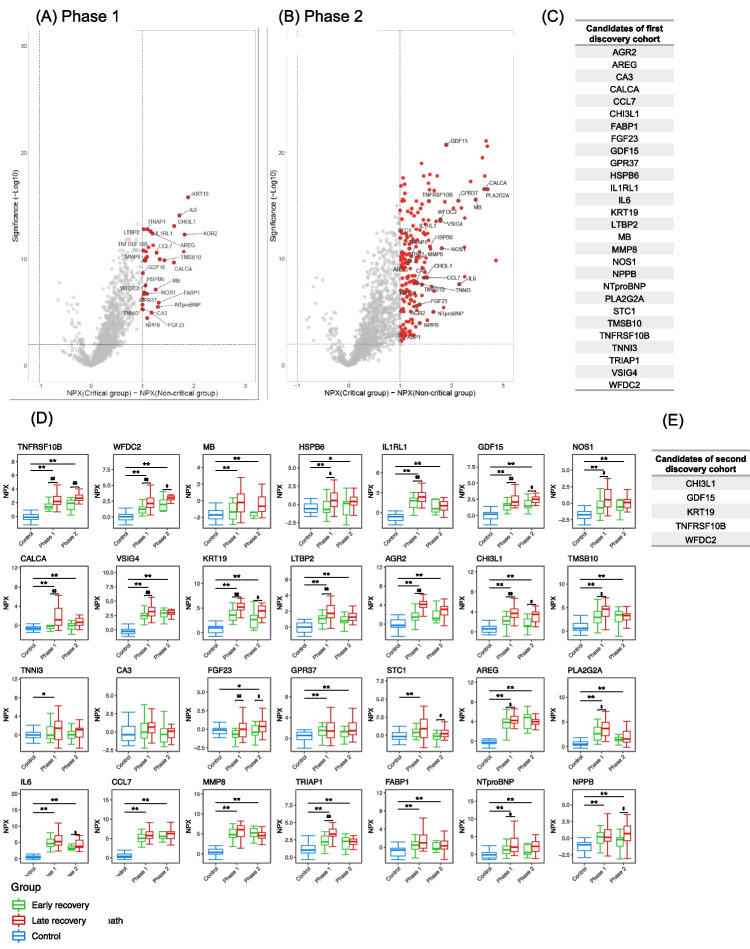
Derivation of candidates’ proteins from the first and second discovery cohorts. Volcano plots show the differentially expressed plasma proteins between critical (acuity max score: A1 or A2) and non-critical (A3, A4, or A5) groups in **A** phase 1 and **B** phase 2 in the first discovery cohort. The *X* axis represents the difference in normalized protein expression (NPX) between the critical group and non-critical group, and the *Y* axis represents log10 significance (adjusted *P* values). Significantly differentially expressed proteins were defined as proteins with adjusted *P* values < 0.01, |difference|> 1. **C** The 28 proteins labeled by gene names that were significantly increased in both phase 1 and phase 2 are shown as candidates of the first discovery cohort. **D** The 28 candidates of the first discovery cohort were evaluated in the second discovery cohort. The levels of the 28 candidates of the first discovery cohort were compared between healthy and COVID-19 individuals in phase 1 and phase 2. The difference between healthy volunteers and COVID-19 patients was measured by Dunnet test (*P < 0.05; **P < 0.01). The COVID-19 individuals were further classified into two groups, “early recovery” and “late recovery” in phase 1 and phase 2. The NPX values are plotted on the *Y* axes. In all box plots, the boxes show median, upper, and lower quartiles, and the whiskers show 5th to 95th percentiles. The difference between two groups was measured by Wilcoxon rank sum test (^$^*P* < 0.05; ^$$^*P* < 0.01). **E** The five candidates of the second discovery cohort are listed

Patient characteristics of the two discovery cohorts are shown in Table 1. In the publicly available first discovery cohort [13], one of the 306 COVID-19 patients was flagged as an outlier and removed from the final dataset, thus leaving 305 phase 1 samples and 139 phase 2 samples. The cohort comprised 109 critical patients and 197 non-critical patients. The distribution of patients by age group was statistically different between the critical and non-critical patients. Other details of the patients’ characteristics are shown in Suppl. Table S1. Plasma proteins showing statistically significant changes in expression are indicated in red in the volcano plots for each phase (Fig. 2A, B), and those that showed statistically significant changes in expression in both phases 1 and 2 are indicated in Fig. 2C as candidates of the first discovery cohort and are labeled in the volcano plots. We derived 28 plasma protein candidates from the first discovery cohort. Phase 1 NPX values of the 28 proteins were associated with acuity max (Suppl. Fig. S1).

**Table 1:** Clinical and demographic characteristics of COVID-19 patients in derivation and validation cohorts

	First discovery cohort, MGH, USA	Second discovery cohort, Osaka, Japan	Validation cohort, Osaka, Japan	*P* value
	(*n* = 306)	(*n* = 53)	(*n* = 113)	
Male sex, n (%)	162 (52.9)	37 (69.8)	80 (70.8)	0.001
Age, years, median (IQR)	58 (45–75)	73 (62–78)	65 (55–74)	
Age group, *n* (%)				0.001
20–34 years	32 (10.5)	0 (0)	1 (0.8)	
35–49 years	66 (21.6)	3 (5.7)	11 (9.7)	
50–64 years	89 (29.1)	13 (26.4)	44 (38.9)	
65–79 years	65 (21.1)	28 (49.1)	47 (41.6)	
Over 80 years	54 (17.6)	10 (18.9)	10 (8.9)	
Comorbidities, *n* (%)				
Heart disease	48 (16.5)	4 (7.7)	12 (10.6)	0.17
Lung disease	66 (21.1)	10 (19.2)	12 (10.6)	0.03
Kidney disease	41 (14.3)	8 (15.4)	12 (10.6)	0.64
Immunocompromised condition	25 (7.7)	5 (9.6)	4 (3.5)	0.21
Hypertension	146 (48.1)	24 (46.2)	47 (41.6)	0.53
Diabetes	111 (35.4)	25 (48.1)	41 (36.3)	0.25
BMI, kg/m^2^, median (IQR)	29 (26–34)	23 (22–26)	25 (22–28)	
BMI, *n* (%)				< 0.001
0–24.9 kg/m^2^	46 (15.1)	35 (66)	49 (43.4)	
25.0–39.9 kg/m^2^	205 (66.9)	16 (30.2)	58 (51.3)	
≥ 40 kg/m^2^	35 (11.4)	0 (0)	1 (0.8)	
Unknown	20 (6.5)	2 (3.8)	5 (4.4)	
Acuity max score, *n* (%)				
1, 28-day mortality	42 (13.7)	5 (9.6)	16 (14.2)	
2, intubated/Ventilated	67 (21.9)	44 (83)	97 (85.8)	
3, hospitalized, O_2_ required	133 (43.5)	4 (7.5)	0 (0)	
4, hospitalized, no O_2_ required	41 (13.4)	0 (0)	0 (0)	
5, discharged/not hospitalized	23 (7.5)	0 (0)	0 (0)	
Ratio of SARS-CoV-2 alpha variant, %	0 (0)	28 (52.8)	57 (50.4)	
SOFA score, median (IQR)	2 (1–7)	5 (3–6)	5 (3–6)	
Outcome				
28-day mortality, *n* (%)	42 (13.7)	1 (1.9)	12 (10.7)	
Hospital mortality, *n* (%)	42 (13.7)	5 (9.6)	16 (14.2)	0.69

Data are reported as number (percentage) or median (IQR, interquartile range) as appropriate *P* value: for the comparison between each cohort

*BMI* body mass index, *Heart disease* coronary artery disease, congestive heart failure, valvular disease, *Lung disease* asthma, COPD, requiring home O_2_ and any chronic lung condition, *Kidney disease* chronic kidney disease, baseline creatinine > 1.5, *Immunocompromised condition* active cancer, chemotherapy, transplant and immunosuppressant agents, asplenic, *SOFA* Sequential Organ Failure Assessment

These 28 candidates were then evaluated in the second discovery cohort that included 53 COVID-19 patients and 20 healthy volunteers (Suppl. Table S2), among whom 49 COVID-19 patients were critical and 4 were treated in the ICU without intubation (Table 1). The numbers of blood samples obtained in phases 1 and 2 were 49 and 34, respectively. We classified the patients into the early recovery group (*n* = 23) and late recovery group (*n* = 30). The age of the late recovery group was statistically higher than that of the early recovery group. The number of days since onset was not different between the two groups (Suppl. Table S3). The common plasma proteins that were higher in the COVID-19 patients than controls and that were higher in the late recovery group than the early recovery group for phases 1 and 2 were WFDC2, CHI3L1, GDF15, KRT19, and TNFRSF10B (Fig. 2D). Expression of these five proteins was higher than that in the control soon after onset, and a correlation between protein expression and the number of days since onset was not clear (Suppl. Fig. S2A). These five proteins in late recovery patients tended to remain high (Suppl. Fig. S2B). We derived these five proteins as candidates of the second discovery cohort (Fig. 2E).

### Network Analysis of 1463 Plasma Proteins in Critical COVID-19 Patients in the Second Discovery Cohort

**Fig. 3:**
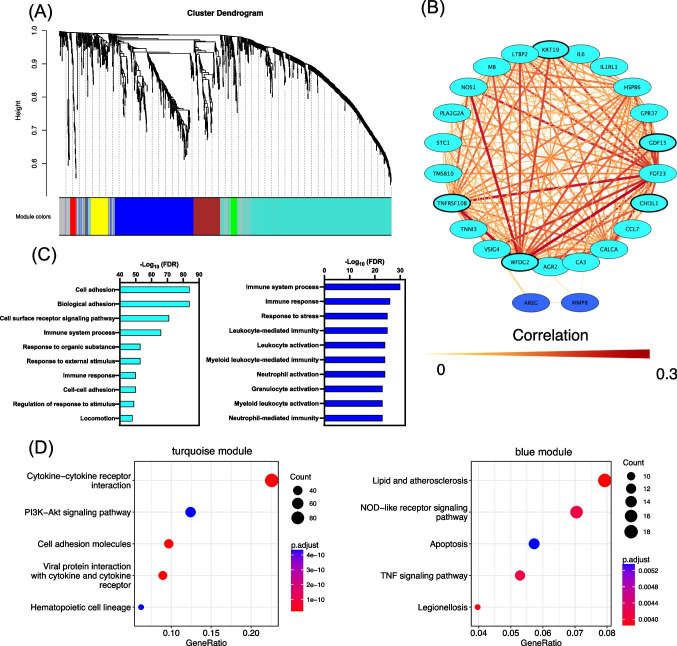
Protein co-expression network in phase 1 of the second discovery cohort. **A** The hierarchical cluster tree of all proteins in the proteomic dataset on the basis of topological overlap. Modules correspond to branches of the tree. The branches and module proteins are colored, and gray indicates proteins outside the appropriate module, as can be seen from the color bands at the bottom of the tree. **B** Network depiction of protein co-expression modules of the 24 candidates of the first discovery cohort. Nodes represent proteins, and edges (lines) indicate connections between the nodes. The color of the nodes corresponds to the module, and the width and color of the edges correspond to the weight of the connected nodes. Bolded nodes indicate the five candidates of the second discovery cohort. **C** Gene ontology enrichment analysis of differentially expressed proteins of the turquoise and blue modules in **A**; the top 10 ontologies for each module are shown. Significantly enriched gene ontology terms are shown with Benjamin-Hochberg false discovery rate–corrected *P* values. **D** KEGG pathway analysis of differentially expressed proteins of the turquoise and blue modules in **A**

In total, six modules were identified (Fig. 3A). Twenty-five of the 28 candidates of the first discovery cohort were included in the turquoise module, as were all five candidates of the second discovery cohort. The 28 candidates of the first discovery cohort were reconstructed and visualized using cytoscape [18] (Fig. 3B). The top 10 GO results for the turquoise and blue modules are shown in Fig. 3C, and the top 5 KEGG results for the turquoise and blue modules are shown in Fig. 3D. The turquoise module is highly related to cell adhesion and biological adhesion. In this analysis, the five candidates of the second discovery cohort were associated with each other, and all had a function involving cell adhesion. The details are shown in the Supplemental Methods, Statistical analysis, and Results (Suppl. Fig. S3). The KEGG pathway of cell adhesion molecules is shown in Suppl. Fig. S4.

### Validation of Five Candidate Plasma Proteins by ELISA

**Fig. 4:**
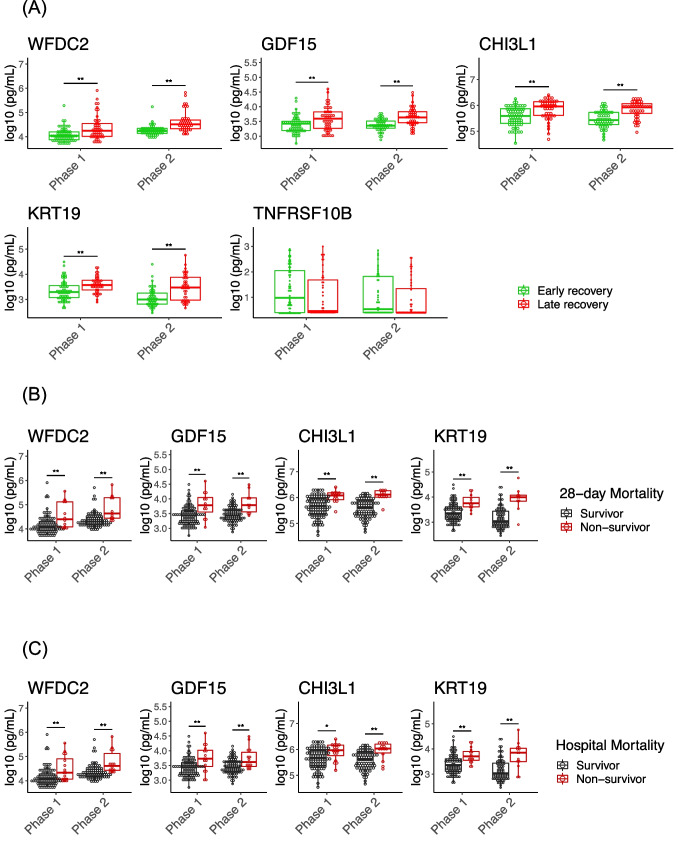
Discovery of four key proteins related to mortality and clinical outcome from the validation cohort. **A** The levels of WFDC2, GDF15, CHI3L1, KRT19, and TNFRSF10B associated with early recovery and late recovery or death in each phase in the COVID-19 groups. **B** The WFDC2, GDF15, CHI3L1, and KRT19 levels for 28-day survivors and non-survivors on each day in the COVID-19 groups. **C** The WFDC2, GDF15, CHI3L1, and KRT19 levels for hospital survivors and non-survivors on each day in the COVID-19 groups. The protein levels were transformed to common logarithmic values to normalize the data distribution. In all box plots, the boxes show median, upper, and lower quartiles, and the whiskers show 5th to 95th percentiles. Asterisks indicate a statistically significant difference (*P < 0.05, **P < 0.01) between two groups on each day by Wilcoxon rank sum test

We assessed the candidate proteins in the validation cohort by ELISA. The validation cohort comprised 113 critical COVID-19 patients including 12 28-day non-survivors, and 16 healthy volunteers. The difference in age between the COVID-19 patients and healthy volunteers was not statistically significant. (Suppl. Table S4). The late recovery group was characterized by older patients, higher D-dimer, creatinine and LDH levels, and lower P/F ratio than those of the early recovery group. The number of days since onset was not different between the two groups (Suppl. Table S5). The numbers of blood samples collected in phases 1 and 2 were 113 and 110, respectively. The levels of WFDC2, GDF15, CHI3L1, and KRT19 were statistically significantly higher in both the COVID-19 patients compared to the controls (Suppl. Fig. S5) and in the late recovery group compared to the early recovery group (Fig. 4A) in both phases. The higher levels of WFDC2, GDF15, CHI3L1, and KRT19 were more frequently observed in the 28-day or hospital non-survivors than in the 28-day or hospital survivors, respectively, in both phases (Fig. 4B, C). There were no relationships between WFDC2, GDF15, CHI3L1, and KRT19, and sex and comorbidities for the control (Suppl. Fig. S6A, B). Only KRT19 of the control was associated with BMI (Suppl. Fig. S6C). GDF15, WFDC2, and CHI3L1 were associated with age in the control and COVID-19 patients (Suppl. Fig. S7). WFDC2, GDF15, CHI3L1, and KRT19 were elevated in the patients who were treated without IMV (Suppl. Table S6, Suppl. Fig. S8). We thus concluded that WFDC2, GDF15, CHI3L1, and KRT19 were four key proteins related to COVID-19 severity.

### Identification of New Clinical Phenotypes Using Latent Cluster Analysis

**Fig. 5:**
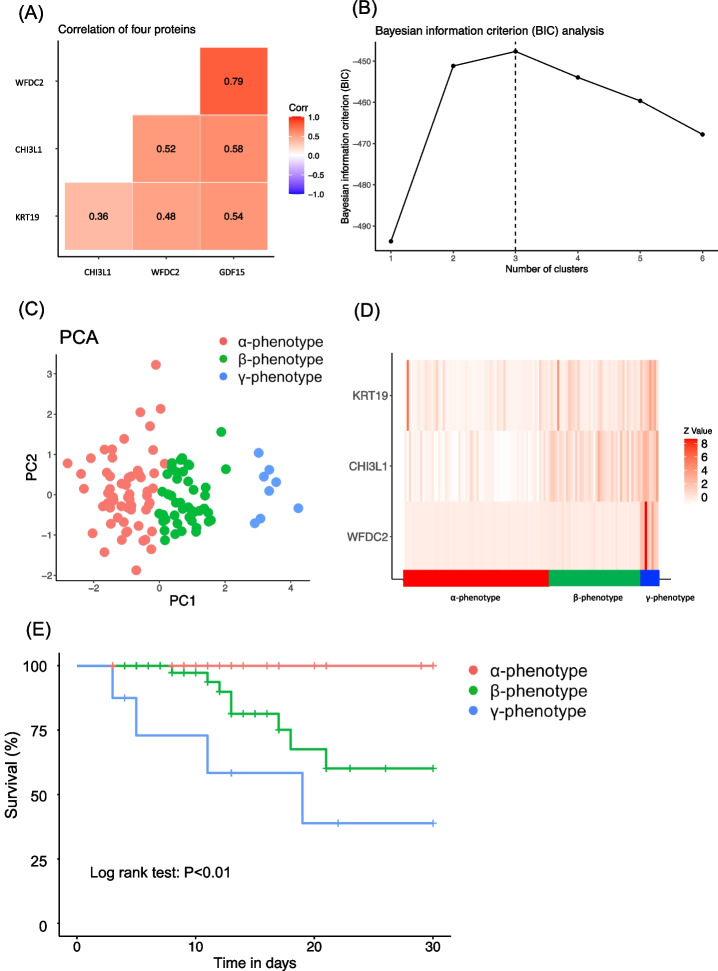
Latent class analysis based on key proteins in the validation cohort. **A** The correlations of WFDC2, CHI3L1, and KRT19 are visualized by heat map. The numbers indicate the Pearson correlation. **B** BIC analysis with the number of clusters on the *X* axis. The BIC was highest for the three-class model. The latent class analysis calculation was performed using the VarSeILCM package in R, where the largest BIC is interpreted as optimal. **C** Visualization of phenotypes using principal component analysis in the validation data. **D** Heat map indicating the impact of the levels of the three proteins (WFDC2, CHI3L1, and KRT19) on the three phenotypes. White signifies the lowest and red the highest *Z*-score. The actual cytokine levels are transformed to *Z*-scores. **E** Kaplan–Meier curve of 28-day survival stratified by latent class analysis-derived phenotype. The log rank test showed significant differences between the three phenotypes (*P* < 0.01)

There were no missing data for the levels of key proteins. A high correlation coefficient (> 0.6) was observed between GDF-15 and WFDC2 (Fig. 5A). WFDC2 can be measured as a tumor marker in Japan, and thus, GDF15 was excluded and WFDC2, CHI3L1, and KRT19 were used as the variables of classification. The BIC was highest for a three-class model and afterwards decreased in proportion to the number of added classes, suggesting that additional classes do not add substantial information to the model (Fig. 5B). The clinical phenotypes were named the *α*, *β*, and *γ* phenotypes. These phenotypes are visualized with principal component analysis in Fig. 5C. The relationships between the clinical phenotypes and the levels of WFDC2, CHI3L1, and KRT19 are visualized in Fig. 5D. The *γ* phenotype showed high levels of all three proteins. The log rank test indicated significant differences between the survival curves among the phenotypes (Fig. 5E). The associations of the clinical phenotypes with clinical data are shown in Suppl. Table S7. The *γ* phenotype was characterized by high creatinine and D-dimer levels and was associated with 28-day and hospital mortality (Suppl. Table S7).

## Discussion

Our study showed that four key proteins, WFDC2 (WAP four-disulfide core domain protein 2, also known as human epididymis protein 4 [HE4]) [26, 27], GDF-15 (growth differentiation factor 15) [28], CHI3L1 (chitinase-3 like-protein-1, also known as YKL-40) [29], and KRT19 (keratin, type I cytoskeletal 19) [30], were associated with the prognosis of COVID-19, and this is supported by the previous reports.

WFDC2 is highly expressed in ovarian cancer [31], systemic sclerosis–related interstitial lung disease [32] and lung adenocarcinoma [33]. It is also expressed in some epithelial cells of the upper airways, mucous cells, and ducts of the submucosal glands and is thought to be involved in innate immunity of the mucosal oral cavity and nasopharynx [26]. Previous reports have shown an association between the severity and prognosis of COVID-19 and WFDC2 [26, 27].

GDF-15 is a member of the transforming growth factor-β molecule superfamily [34] and is highly expressed in macrophages, airway epithelial cells, and vascular endothelial cells [35]. It has been reported to be an independent prognostic factor in cardiovascular disease, lung disease, and sepsis [36–38]. Several reports show the association between the levels of GDF-15 and disease severity in COVID-19 [28, 39]. As mentioned above, GDF-15 is well observed to be upregulated under stress conditions, but the mechanism for this is unclear. Further research is needed, including that into which tissues express GDF-15 in COVID-19.

CHI3L1 is a member of glycoside hydrolase family 18 and is synthesized and secreted by many cells, including macrophages, neutrophils, synoviocytes, smooth muscle cells, and tumor cells [40]. CHI3L1 has been reported to promote cancer growth, production of proinflammatory cytokines, and microglial activation [41]. It is strongly associated with diseases such as asthma, arthritis, sepsis, diabetes, liver fibrosis, and coronary artery disease [29]. In COVID-19, CHI3L1 is reported to be associated with severe disease, although it is not correlated with mortality. It is suggested that CHI3L1 is a major stimulator of ACE2, promotes binding and activation of SC2 S-protein-receptor, and enhances infection and the spread of COVID-19 [29, 42]. In the present study, GDF15, WFDC2, and CHI3LI were correlated with age in the COVID-19 patients. It has been reported that the levels of circulating CHI3L1 increase with aging in healthy controls, whereas levels of circulating CHI3L1 increase in patients with severe COVID19 compared to healthy controls regardless of age [29].

KRT19 is one of the most important cytokeratins expressed in epithelial and mesothelial tissues, and its overexpression has been reported in more than 30 malignant neoplasms, including lung and breast cancer [43]. Cyfra21-1 proteins, a fragment of KRT19, have been reported to be useful among lung cancers as a marker for non-small cell lung cancer (squamous cell carcinoma) [44]. In COVID-19, although Gisby et al. [45] reported the association of severity with upregulation of KRT19 using a proteomics approach, there are still few COVID-19-related reports on this cytokeratin.

Our previous studies showed that the key cytokine proteins formed a cytokine network in the patients including those with sepsis and burn [7–9]. In the present study, WGCNA revealed six protein network clusters. The GO enrichment and KEGG pathway analyses for each cluster indicated that the four key proteins are mainly involved in clusters related to cell adhesion pathways in addition to previously reported immune responses [46, 47]. This suggests that the four key proteins interact with each other in the cell adhesion pathways, which may play a key role in the pathogenesis of critical COVID-19.

The immune response associates with a complex interaction of factors involving comorbidities, age, weight, sex, ethnic background, pathogen types, and environment in the patients, thus resulting in a heterogeneous disease phenotype. The phenotype also varies between individuals over the time course of the disease. Mathew et al. showed that based on high-dimensional cytometry information, three immunotypes were associated with poor clinical trajectories in COVID-19 patients [47]. Also, Shu et al. distinguished different severity using LC–MS/MS on the basis of machine-learning models [48]. In our study, the critical COVID-19 patients were divided into three phenotypes (*α*, *β*, *γ*) using WFDC2, CHI3L1, and KRT19 on day 1 by latent class analysis. Patients with the *β* and *γ* phenotypes had lower survival rates and more prolonged ventilation times than those with the *α* phenotype, indicating that the *β* and *γ* phenotypes could be potential therapeutic targets for intervention in critical COVID-19.

This study has several limitations. First, age could have affected the plasma proteins levels. The difference in ages between the critical and non-critical patients in the first discovery cohort, between the control and COVID-19 patients, and between patients with early and late recovery in the second discovery cohort could have affected the process to derive the five candidate proteins. Second, the phase was defined as the time from visiting the emergency department or admission to the hospital, and thus, the time from onset was not considered. It was not clear what triggers the protein elevation, when the protein elevation occurs and how long the proteins elevation continued; therefore, the possibility of missing important proteins due to focusing on specific periods, phase 1 and phase 2, remains. However, in clinical practice, the time of infection varies as the time of admission to the emergency department or ICU, and this study may be more relevant to actual clinical practice. Third, we used three cohorts that included different variables. Therefore, information on unmeasured confounders and treatment details is lacking that may have biased the results. Fourth, basic treatment strategies of the participating facilities may have differed in their details. Such variation between the treatment centers could slightly influence the levels of proteins and the findings in this analysis. Finally, we did not perform a validation of the clinical phenotypes and the prediction model in another cohort.

## Conclusion

The use of a new plasma proteomics approach revealed four key proteins in the blood validated by ELISA that were associated with COVID-19 pathogenesis. The clinical phenotypes based on WFDC2, CHI3L1, and KRT19 were significantly associated with patient prognosis.

## Supplementary Information

Below is the link to the electronic supplementary material.

Supplementary file1 Association between 28 candidates of phase1 and Acuitymax scores. The phase 1 normalized protein expression (NPX) values of thecandidates were compared among Acuity max scores by Kruskal-Wallis test. Acuitymax scores are indicated as follows: A1, Dead; A2, Intubated; A3, Oxygentherapy; A4, No oxygen therapy; A5, Discharged from ED. *ED* emergencydepartment. Asterisks indicate a statistically significant difference (*P <0.05, **P < 0.01). (PDF 697 KB)

Supplementary file2 Correlation betweennormalized protein expression (NPX) values of 28 candidates and the number ofdays since onset. (A) The NPX values of controls were also plotted on the leftside in the figure (orange color). The data include phase 1 and phase 2 data.Correlation analysis was performed using Spearman’s rank correlation analysis.(B) The NPX values of early recovery are colored in green and those of laterecovery are colored in red. Linear regressions (solid lines) with 95% confidenceintervals (gray areas) are shown (PDF 590 KB)

Supplementary file3 Protein co-expression network analysis for criticalCOVID-19 by use of weighted gene co-expression network analysis. (A)Sample dendrogram of 38 samples and clinical trait heatmap. (B) Analysis ofnetwork topology for various soft-threshold powers. (C) Analysis of meanconnectivity as a function of the soft-threshold power. (D) Visualization ofthe network using a heatmap plot. The heatmap depicts topological overlaps,with light colors denoting low overlap and darker colors higher adjacencyoverlap. The dendrogram and module assignments are shown along the left sideand the top. (E) Heatmap of the correlation between the module and clinicaltraits. Each cell contains the corresponding correlation and P value. The tableis color-coded by correlation according to the color legend (PDF 16641 KB)

Supplementary file4 KEGG pathway map of CELLADHESION MOLECULES. Proteins in red are included in the turquoise module. *KEGG*Kyoto Encyclopedia of Genes and Genomes. (PDF 149 KB)

Supplementary file5 Change in the levels of the five candidates of the second discoverycohort compared between healthy and COVID-19 patients.The levels of the candidates weretransformed to common logarithmic values to normalize the data distribution. Inall box plots, the boxes show median, upper and lower quartiles, and thewhiskers show 5th to 95th percentiles. * or ** indicates a significantdifference in proteins between control and COVID-19 patients on each day byDunnet test (*P < 0.05; **P < 0.01). (PDF 173 KB)

Supplementary file6 Associationbetweenthe four key proteins and sex, comorbidities and body massindex (BMI). Thelevels of the four key proteins were transformed to common logarithmic valuesto normalize the data distribution. (A) The four proteins of the control and phase 1of the COVID-19 patients were compared between sexes by Wilcoxon rank sum test.In all box plots, the boxes show median, upper and lower quartiles, and thewhiskers show 5th to 95th percentiles. * or ** indicates a significant differencein proteins between control and COVID-19 patients (*P < 0.05; **P < 0.01)(B) The four proteins of the control and phase 1 of the COVID-19 patient werecompared between comorbidities by Wilcoxon rank sum test. In all box plots, theboxes show median, upper and lower quartiles, and the whiskers show 5th to 95thpercentiles. * or ** indicates a significant difference in proteins betweencontrol and COVID-19 patients (*P < 0.05; **P < 0.01) (C) Correlationbetween the four proteins and BMI. Spearman’s correlation was used to evaluate the correlation between theprotein levels and BMI. (PDF 567 KB)

Supplementary file7 Correlationbetween the four proteins and age. Spearman’scorrelation was used to evaluate the correlation between the protein levels andage. (PDF 119 KB)

Supplementary file8 Association between the five candidates of the validation cohort and COVID-19 patients treated without invasive mechanical ventilation (IMV). The levels of the five key proteins were transformed to common logarithmic values to normalize the data distribution. The five proteins of the control and the COVID-19 patients treated without IMV and with IMV were compared by Wilcoxon rank sum test with Bonferroni correction. In all box plots, the boxes show median, upper and lower quartiles, and the whiskers show 5th to 95th percentiles. * or ** indicates a significant difference in proteins between control and COVID-19 patients (*P < 0.05; **P < 0.01). $ or $$ indicates a significant difference in proteins between COVID-19 treated without IMV and with IMV ($P < 0.05; $$P < 0.01) (page 42, lines 730-739). (PDF 75 KB)

Supplementary file9 (DOCX 21 KB)

Supplementary file10 (DOCX 19 KB)

Supplementary file11 (DOCX 26.5 KB)

Supplementary file12 (DOCX 21 KB)

Supplementary file13 (DOCX 25.2 KB)

Supplementary file14 (DOCX 21 KB)

Supplementary file15 (DOCX 22 KB)

## Data Availability

Original Olink proteomics data have been deposited to Mendeley Data: 10.17632/2cbxgsn7vx.1.

## Abbreviations

BIC: Bayesian information criterion
CHI3L1: Chitinase-3 like-protein-1
COVID-19: Coronavirus disease 2019
ELISA: Enzyme-linked immunosorbent assay
GDF15: Growth differentiation factor 15
ICU: Intensive care unit
IMV: Invasive mechanical ventilation
KRT19: Keratin, type I cytoskeletal 19
NPX: Normalized protein expression
TNFRSF10B: TNF Receptor Superfamily Member 10b
WFDC2: WAP four-disulfide core domain protein 2
WGCNA: Weighted gene co-expression network analysis
