# Supplementary figures and images for: Combination of WFDC2, CHI3L1, and KRT19 in Plasma Defines a Clinically Useful Molecular Phenotype Associated with Prognosis in Critically Ill COVID-19 Patients
Source: J Clin Immunol. 2022 Nov 4;43(2):286–98. doi: 10.1007/s10875-022-01386-3 (PMC9638294; doi:10.1007/s10875-022-01386-3)

Supplemental Figure 1.

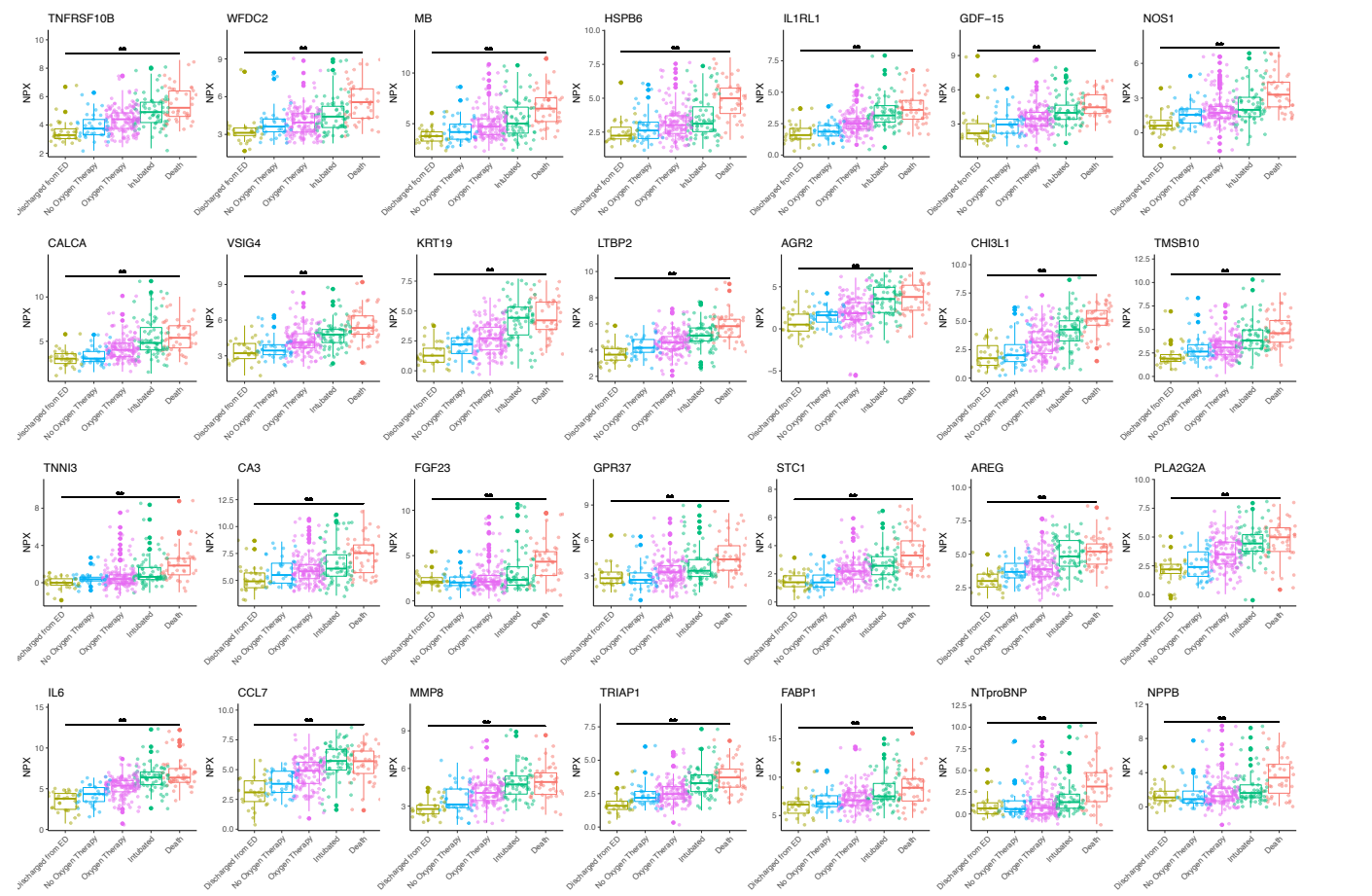

Supplement: Supplementary file 1 — Supplementary file1 Association between 28 candidates of phase1 and Acuitymax scores. The phase 1 normalized protein expression (NPX) values of thecandidates were compared among Acuity max scores by Kruskal-Wallis test. Acuitymax scores are indicated as follows: A1, Dead; A2, Intubated; A3, Oxygentherapy; A4, No oxygen therapy; A5, Discharged from ED. ED emergencydepartment. Asterisks indicate a statistically significant difference (*P <0.05, **P < 0.01). (PDF 697 KB) [file 10875_2022_1386_MOESM1_ESM.pdf]

# Supplemental Figure 2.

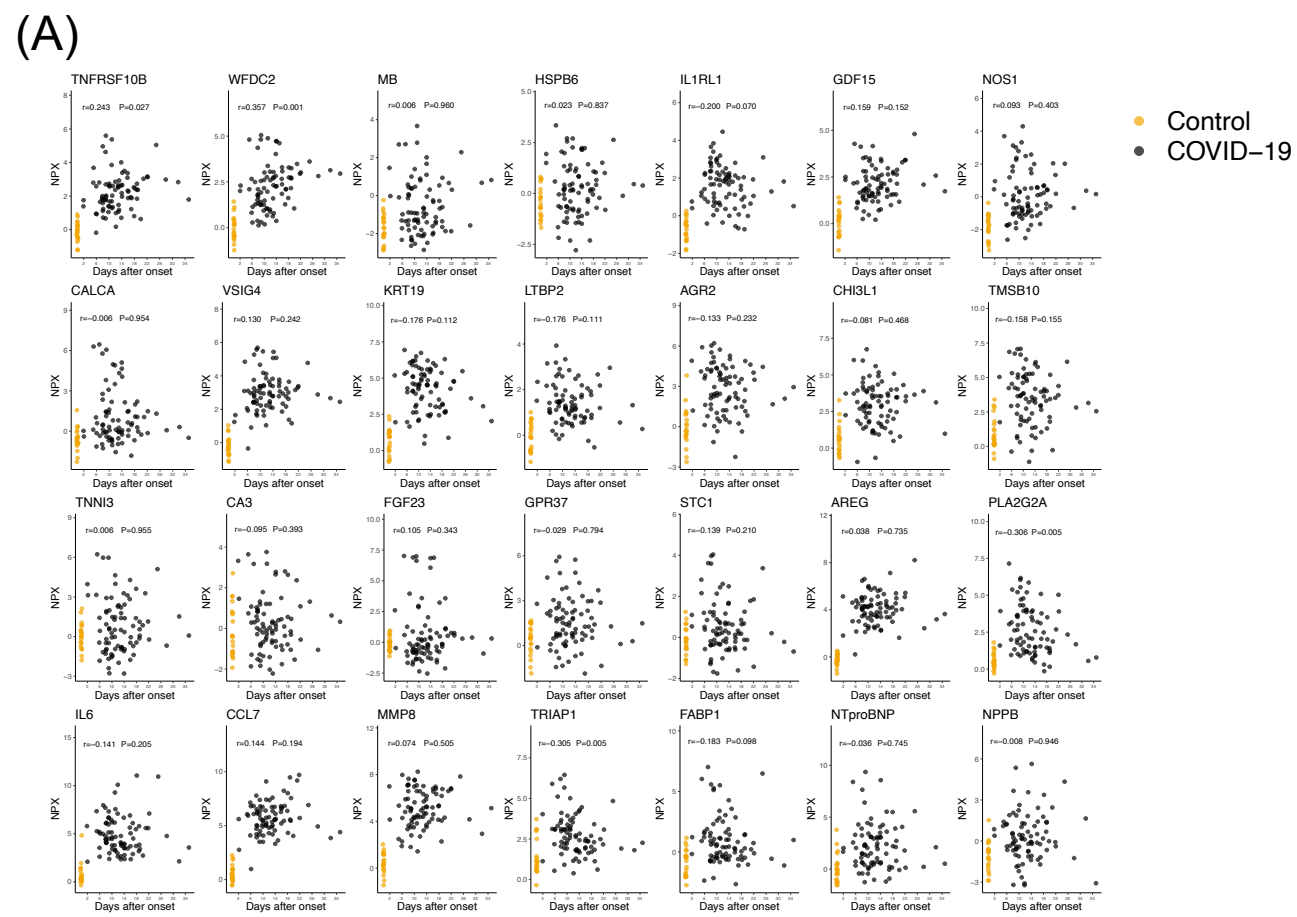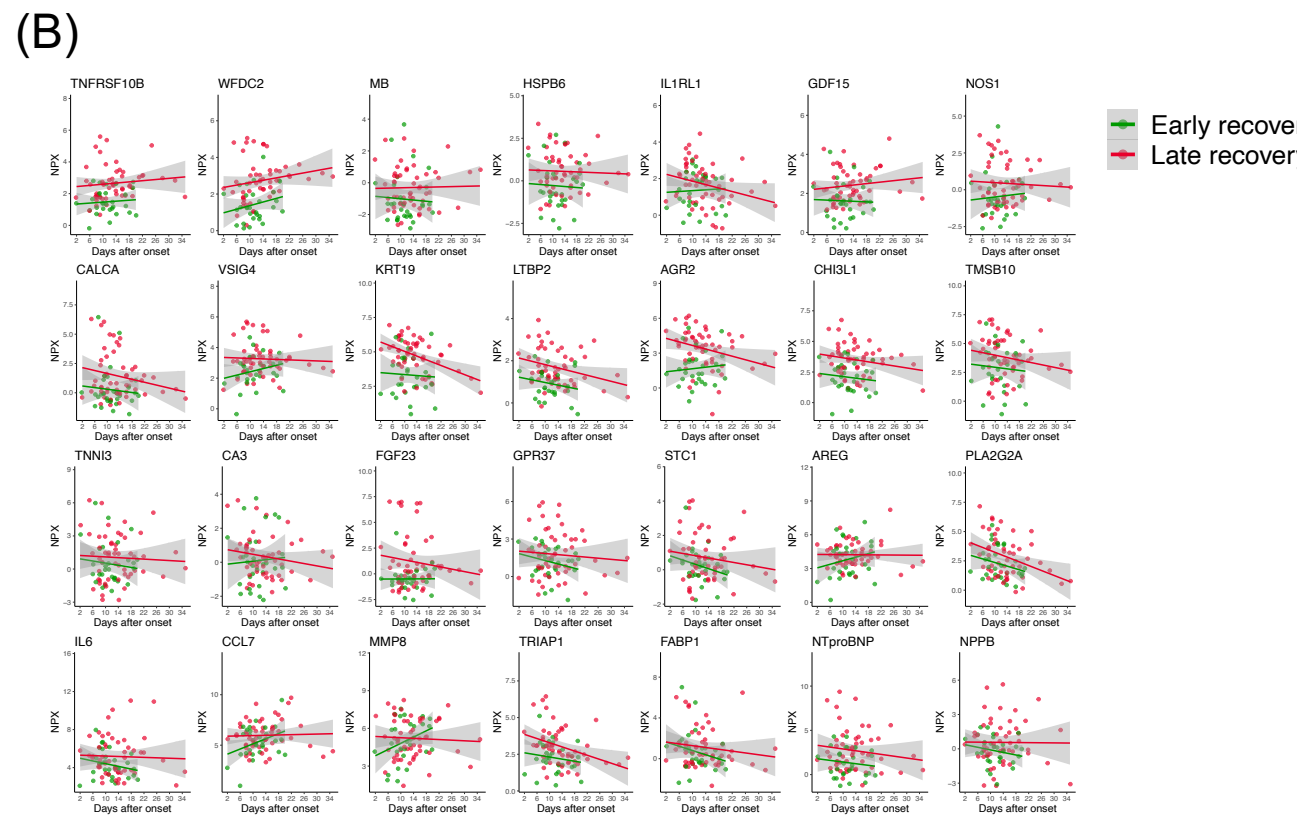

Supplement: Supplementary file 2 — Supplementary file2 Correlation betweennormalized protein expression (NPX) values of 28 candidates and the number ofdays since onset. (A) The NPX values of controls were also plotted on the leftside in the figure (orange color). The data include phase 1 and phase 2 data.Correlation analysis was performed using Spearman’s rank correlation analysis.(B) The NPX values of early recovery are colored in green and those of laterecovery are colored in red. Linear regressions (solid lines) with 95% confidenceintervals (gray areas) are shown (PDF 590 KB) [file 10875_2022_1386_MOESM2_ESM.pdf]

Supplemental Figure 3.

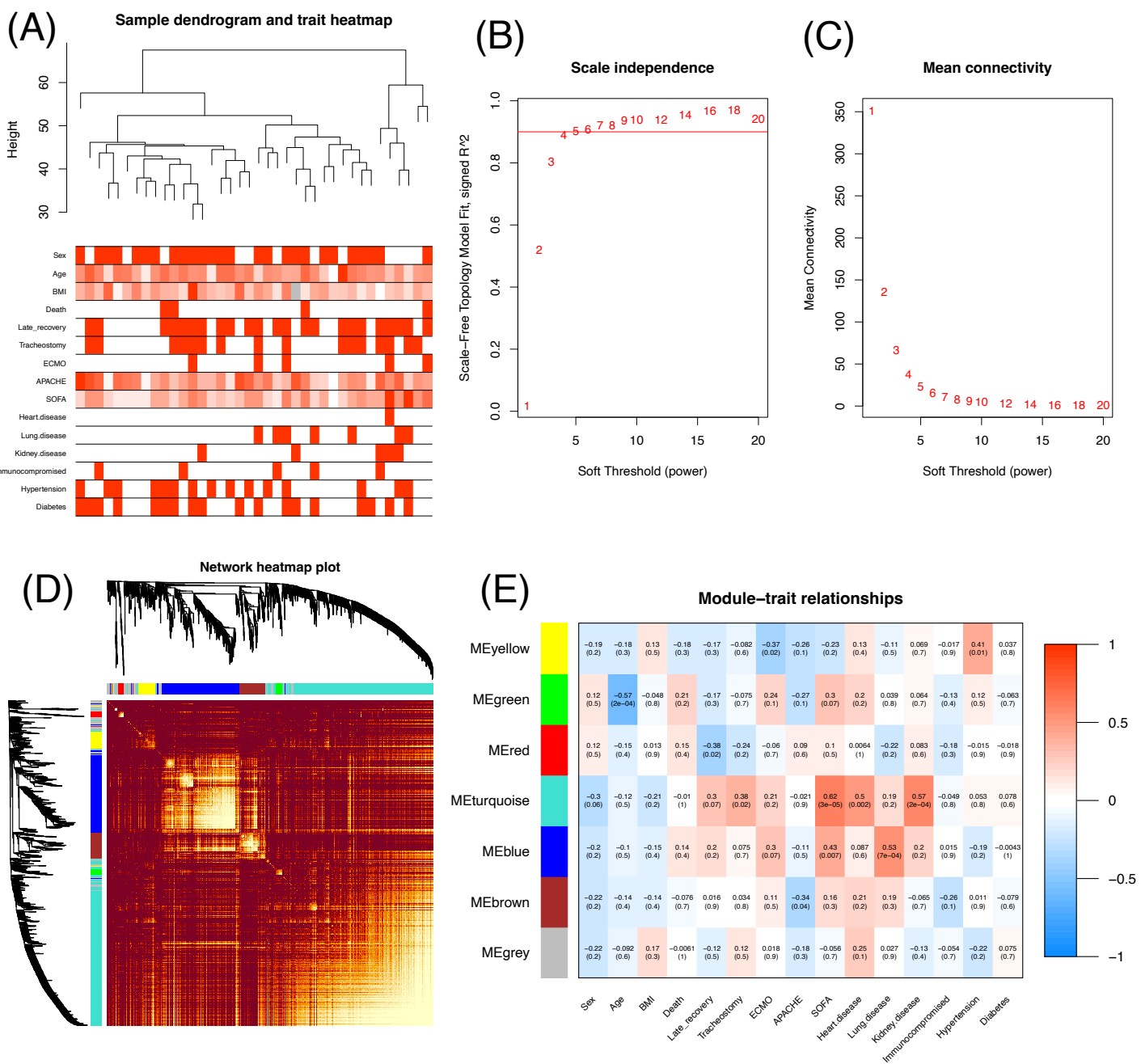

Supplement: Supplementary file 3 — Supplementary file3 Protein co-expression network analysis for criticalCOVID-19 by use of weighted gene co-expression network analysis. (A)Sample dendrogram of 38 samples and clinical trait heatmap. (B) Analysis ofnetwork topology for various soft-threshold powers. (C) Analysis of meanconnectivity as a function of the soft-threshold power. (D) Visualization ofthe network using a heatmap plot. The heatmap depicts topological overlaps,with light colors denoting low overlap and darker colors higher adjacencyoverlap. The dendrogram and module assignments are shown along the left sideand the top. (E) Heatmap of the correlation between the module and clinicaltraits. Each cell contains the corresponding correlation and P value. The tableis color-coded by correlation according to the color legend (PDF 16641 KB) [file 10875_2022_1386_MOESM3_ESM.pdf]

### Supplemental Figure 4.

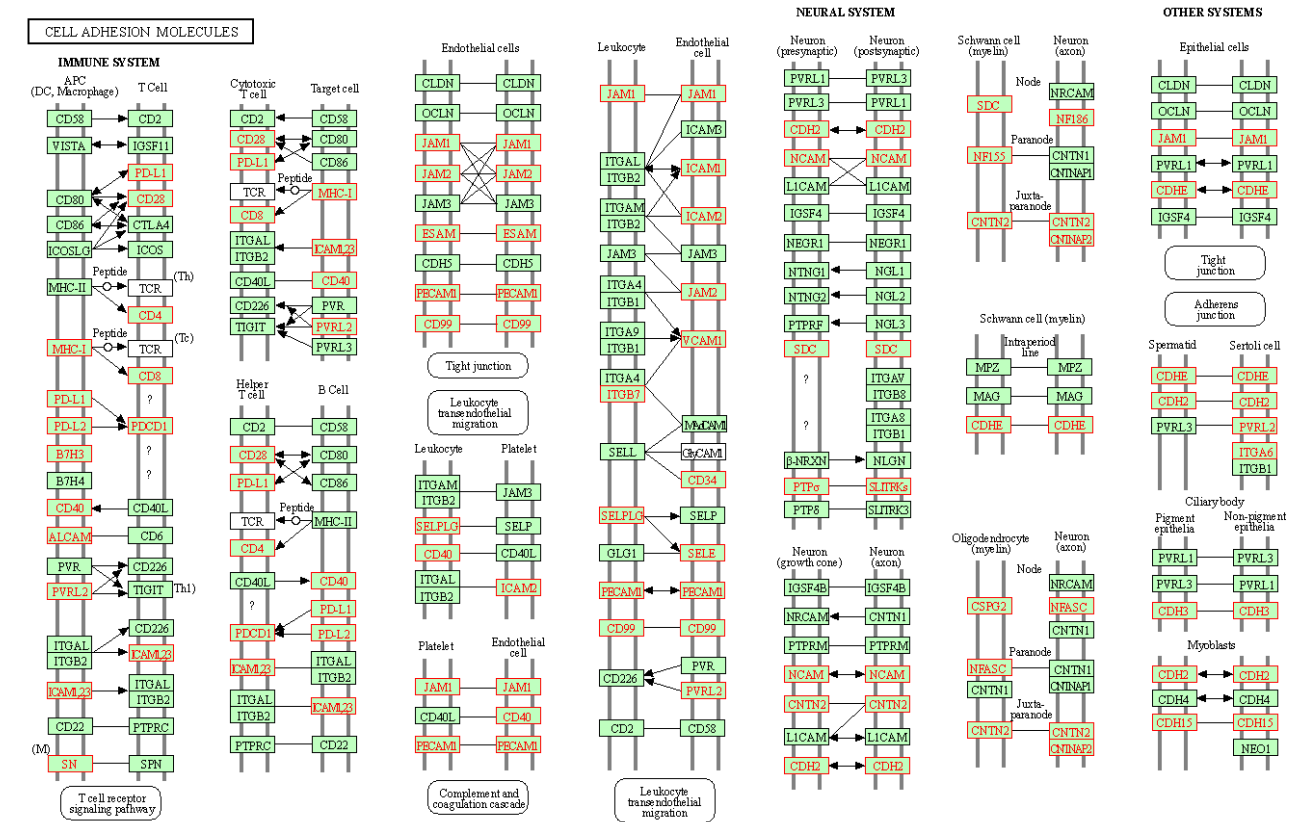

Supplement: Supplementary file 4 — Supplementary file4 KEGG pathway map of CELLADHESION MOLECULES. Proteins in red are included in the turquoise module. KEGGKyoto Encyclopedia of Genes and Genomes. (PDF 149 KB) [file 10875_2022_1386_MOESM4_ESM.pdf]

Supplemental Figure 5.

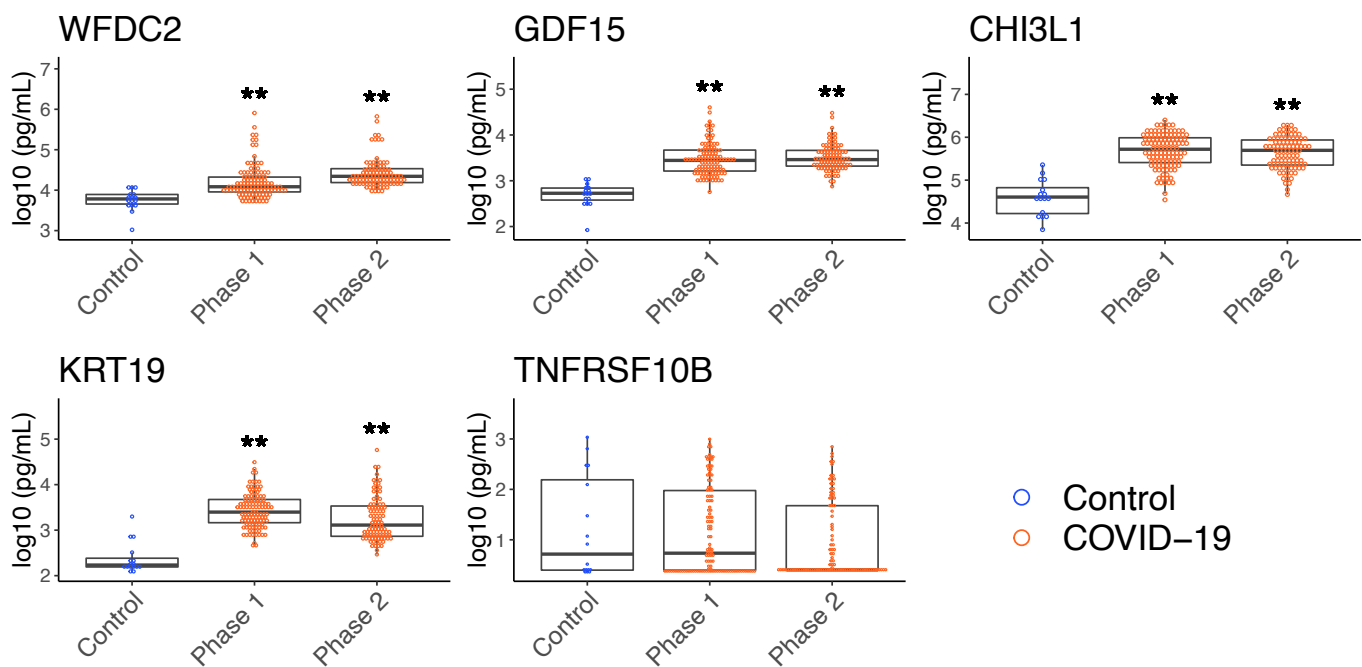

Supplement: Supplementary file 5 — Supplementary file5 Change in the levels of the five candidates of the second discoverycohort compared between healthy and COVID-19 patients.The levels of the candidates weretransformed to common logarithmic values to normalize the data distribution. Inall box plots, the boxes show median, upper and lower quartiles, and thewhiskers show 5th to 95th percentiles. * or ** indicates a significantdifference in proteins between control and COVID-19 patients on each day byDunnet test (*P < 0.05; **P < 0.01). (PDF 173 KB) [file 10875_2022_1386_MOESM5_ESM.pdf]

Supplemental Figure 6.

(A)

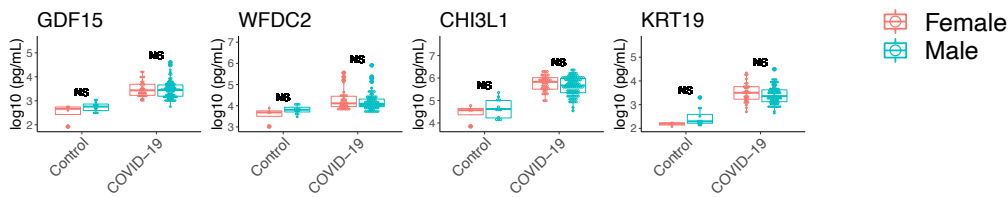

(B)

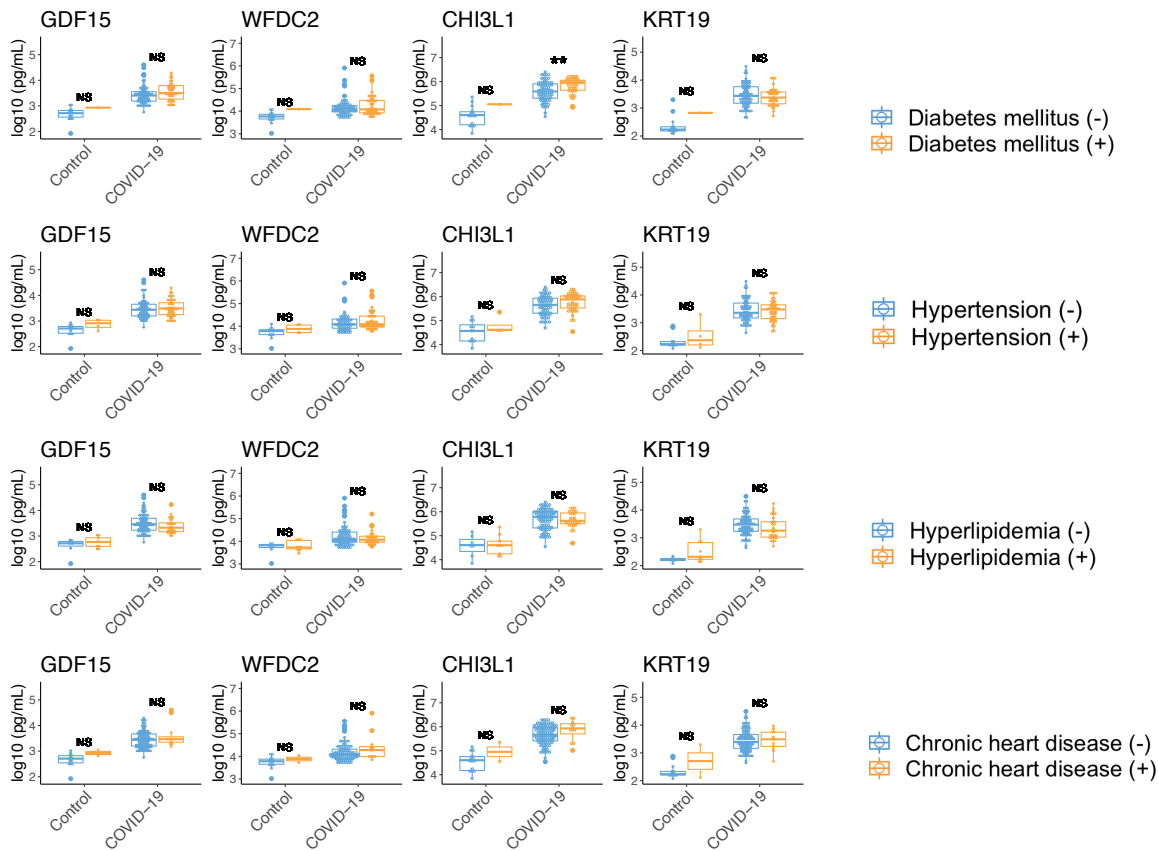

(C)

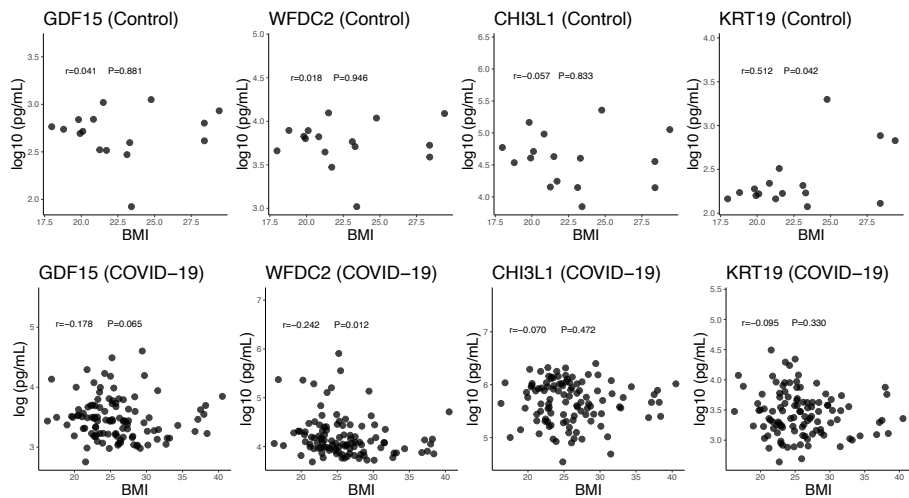

Supplement: Supplementary file 6 — Supplementary file6 Associationbetweenthe four key proteins and sex, comorbidities and body massindex (BMI). Thelevels of the four key proteins were transformed to common logarithmic valuesto normalize the data distribution. (A) The four proteins of the control and phase 1of the COVID-19 patients were compared between sexes by Wilcoxon rank sum test.In all box plots, the boxes show median, upper and lower quartiles, and thewhiskers show 5th to 95th percentiles. * or ** indicates a significant differencein proteins between control and COVID-19 patients (*P < 0.05; **P < 0.01)(B) The four proteins of the control and phase 1 of the COVID-19 patient werecompared between comorbidities by Wilcoxon rank sum test. In all box plots, theboxes show median, upper and lower quartiles, and the whiskers show 5th to 95thpercentiles. * or ** indicates a significant difference in proteins betweencontrol and COVID-19 patients (*P < 0.05; **P < 0.01) (C) Correlationbetween the four proteins and BMI. Spearman’s correlation was used to evaluate the correlation between theprotein levels and BMI. (PDF 567 KB) [file 10875_2022_1386_MOESM6_ESM.pdf]

Supplemental Figure 7.

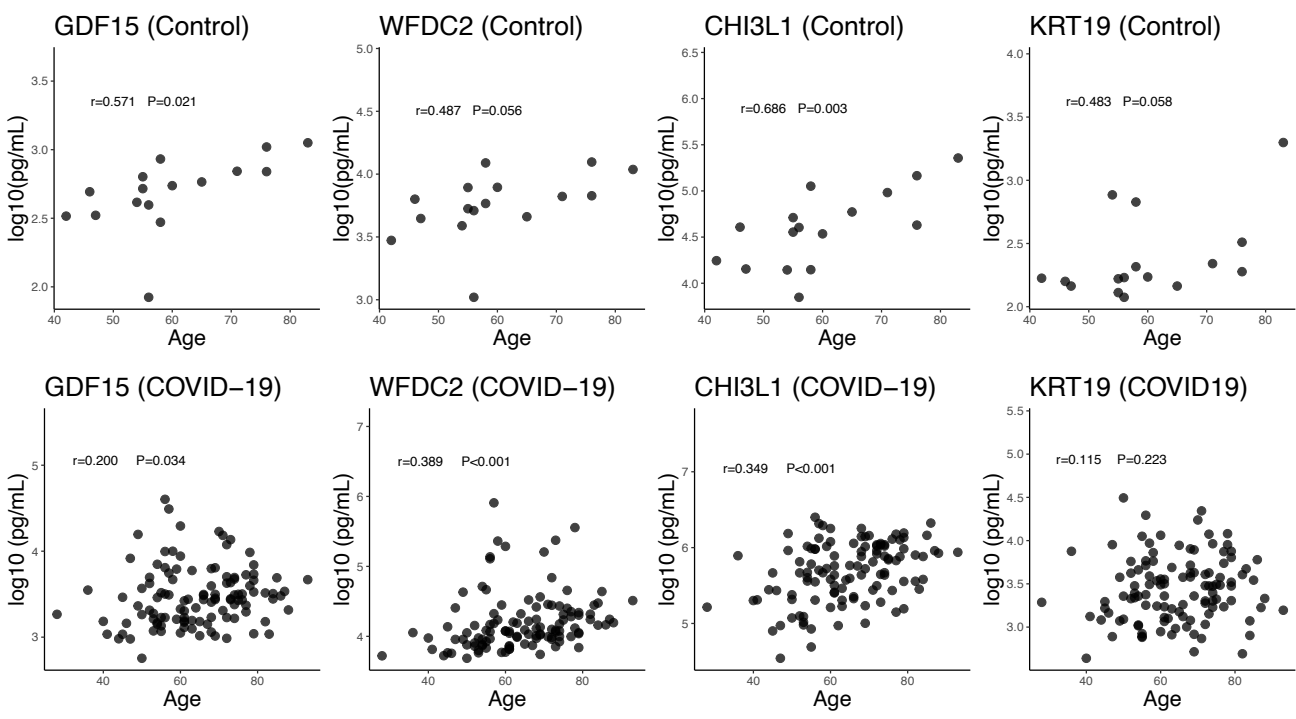

Supplement: Supplementary file 7 — Supplementary file7 Correlationbetween the four proteins and age. Spearman’scorrelation was used to evaluate the correlation between the protein levels andage. (PDF 119 KB) [file 10875_2022_1386_MOESM7_ESM.pdf]

Supplemental Figure 8.

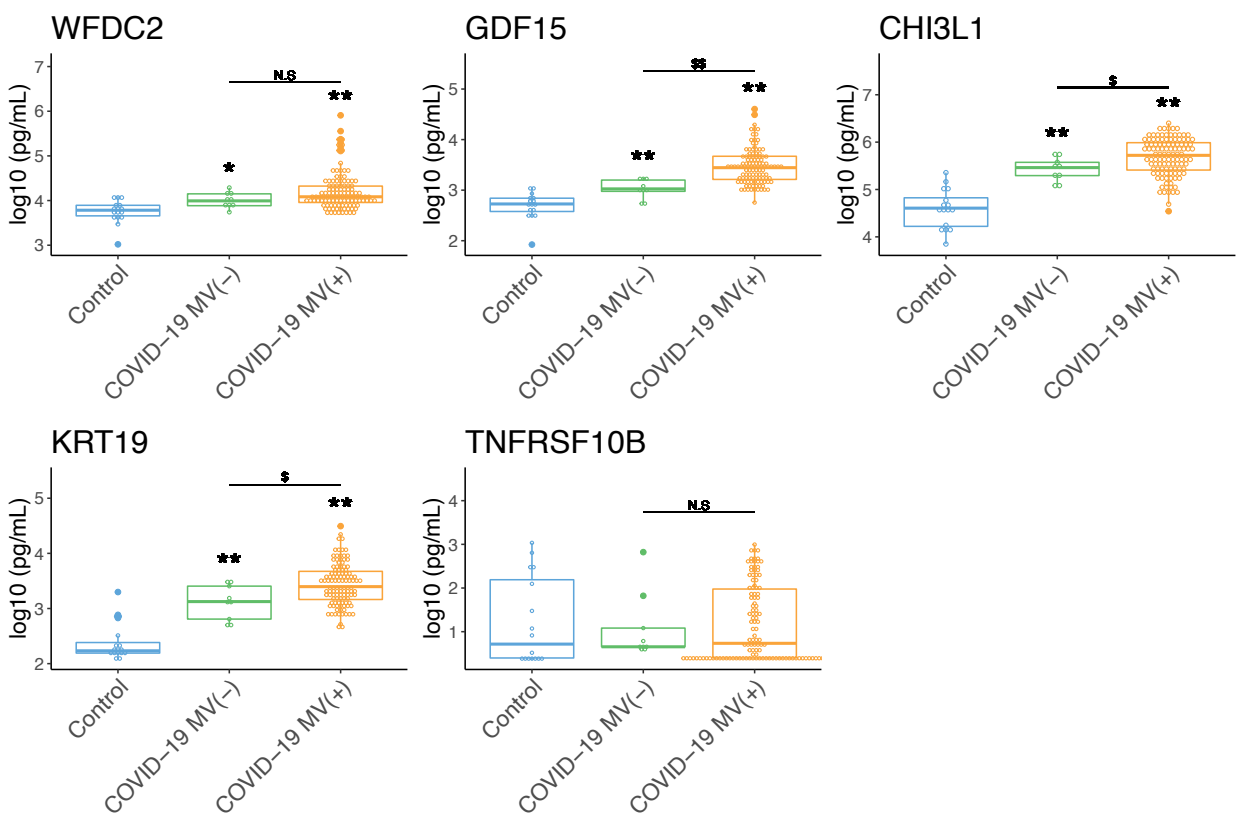

Supplement: Supplementary file 8 — Supplementary file8 Association between the five candidates of the validation cohort and COVID-19 patients treated without invasive mechanical ventilation (IMV). The levels of the five key proteins were transformed to common logarithmic values to normalize the data distribution. The five proteins of the control and the COVID-19 patients treated without IMV and with IMV were compared by Wilcoxon rank sum test with Bonferroni correction. In all box plots, the boxes show median, upper and lower quartiles, and the whiskers show 5th to 95th percentiles. * or ** indicates a significant difference in proteins between control and COVID-19 patients (*P < 0.05; **P < 0.01). $ or $$ indicates a significant difference in proteins between COVID-19 treated without IMV and with IMV ($P < 0.05; $$P < 0.01) (page 42, lines 730-739). (PDF 75 KB) [file 10875_2022_1386_MOESM8_ESM.pdf]
