# Supplementary material for: Combination of WFDC2, CHI3L1, and KRT19 in Plasma Defines a Clinically Useful Molecular Phenotype Associated with Prognosis in Critically Ill COVID-19 Patients
Source: J Clin Immunol. 2022 Nov 4;43(2):286–98. doi: 10.1007/s10875-022-01386-3 (PMC9638294; doi:10.1007/s10875-022-01386-3)
Supplement: Supplementary file 9 — Supplementary file9 (DOCX 21 KB) [file 10875_2022_1386_MOESM9_ESM.docx]

| **Supplemental Table 1** Clinical and demographic characteristics of COVID-19 patients in 1st discovery cohort | | | | |
| --- | --- | --- | --- | --- |
|  | Overall | Critical group | Non-critical group | p Value |
|  | (n=306) | (n=109) | (n=197) |  |
| Male sex, n (%) | 162 (52.9) | 64 (58.7) | 98 (49.7) | 0.12 |
| Age, median years (IQR) | 58 (45-75) |  |  |  |
| Age group, n (%) |  |  |  | <0.001 |
| 20–34 years | 32 (10.5) | 4 (3.7) | 28 (14.1) |  |
| 35–49 years | 66 (21.6) | 14 (12.8) | 52 (26.4) |  |
| 50–64 years | 89 (29.1) | 27 (24.8) | 62 (31.5) |  |
| 65–79 years | 65 (21.1) | 37 (33.9) | 28 (14.1) |  |
| Over 80 years | 54 (17.6) | 27 (24.8) | 27 (13.7) |  |
| Comorbidities, n (%) |  |  |  |  |
| Heart disease | 48 (16.5) | 23 (21.1) | 25 (12.7) | 0.06 |
| Lung disease | 66 (21.1) | 16 (14.7) | 50 (25.4) | 0.03 |
| Kidney disease | 41 (14.3) | 25 (22.9) | 16 (8.1) | <0.001 |
| Immunocompromised condition | 25 (7.7) | 14 (12.8) | 11 (5.6) | 0.03 |
| Hypertension | 146 (48.1) | 65 (59.6) | 81 (41.1) | 0.002 |
| Diabetes | 111 (35.4) | 50 (45.8) | 61 (30.9) | 0.01 |
| BMI, kg/m^2^, median (IQR) | 29 (26-34) |  |  |  |
| BMI, n (%) |  |  |  | 0.21 |
| 0–24.9 kg/m^2^ | 46 (15.1) | 19 (17.4) | 27 (13.7) |  |
| 25.0–39.9 kg/m^2^ | 205 (66.9) | 73 (67.1) | 132 (67) |  |
| Over 40 kg/m^2^ | 35 (11.4) | 13 (11.9) | 22 (11.1) |  |
| Unknown | 20 (6.5) | 4 (3.7) | 16 (8.1) |  |
| Acuity max score |  |  |  | <0.001 |
| 1=28-day mortality | 42 (13.7) | 42 (38.5) | 0 (0) |  |
| 2=Intubated/ventilated, survived | 67 (21.9) | 67 (21.9) | 0 (0) |  |
| 3=Hospitalized, O_2_ required, survived | 133 (43.5) | 0 (0) | 133 (67.5) |  |
| 4=Hospitalized, no O_2_ required, survived | 41 (13.4) | 0 (0) | 41 (20.8) |  |
| 5=Discharged/Not hospitalized, survived | 23 (7.5) | 0 (0) | 23 (11.7) |  |
| Outcome |  |  |  |  |
| 28-day mortality, n (%) | 42 (13.7) | 42 (38.5) | 0 | <0.001 |
| Data are reported as number(percentage) or median (IQR, interquartile range) as appropriate  p Value: for the comparison between critical and noncritical group  *Heart disease* coronary artery disease, congestive heart failure, valvular disease, *Lung disease* asthma, COPD, requiring home O_2_ and any chronic lung condition, *Kidney disease* chronic kidney disease, baseline creatinine >1.5, *Immunocompromised condition* active cancer, chemotherapy, transplant and immunosuppressant agents, asplenic, *BMI* body mass index | | | | |
