# Supplementary material for: Combination of WFDC2, CHI3L1, and KRT19 in Plasma Defines a Clinically Useful Molecular Phenotype Associated with Prognosis in Critically Ill COVID-19 Patients
Source: J Clin Immunol. 2022 Nov 4;43(2):286–98. doi: 10.1007/s10875-022-01386-3 (PMC9638294; doi:10.1007/s10875-022-01386-3)
Supplement: Supplementary file 10 — Supplementary file10 (DOCX 19 KB) [file 10875_2022_1386_MOESM10_ESM.docx]

| **Supplemental Table 2** Clinical and demographic characteristics of COVID-19 patients in 2nd discovery cohort | | | |
| --- | --- | --- | --- |
|  | COVID-19 patient | Healthy volunteers | p Value |
|  | (n=53) | (n=20) |  |
| Male sex, n (%) | 37 (69.8) | 14 (70) | 0.86 |
| Age, median years (IQR) | 73 (62-78) | 59 (55-71) | 0.001 |
| Age group, n (%) |  |  | 0.07 |
| 20–34 years | 0 (0) | 0 (0) |  |
| 35–49 years | 3 (5.7) | 2 (10) |  |
| 50–64 years | 13 (26.4) | 13 (65) |  |
| 65–79 years | 28 (49.1) | 5 (25) |  |
| Over 80 years | 10 (18.9) | 0 (0) |  |
| Comorbidities, n (%) |  |  |  |
| Heart disease | 4 (7.7) | 2 (10) | 0.75 |
| Lung disease | 10 (19.2) | 0 (0) | 0.01 |
| Kidney disease | 8 (15.4) | 0 (0) | 0.02 |
| Immunocompromised condition | 5 (9.6) | 0 (0) | 0.15 |
| Hypertension | 24 (46.2) | 4 (20) | 0.04 |
| Diabetes | 25 (48.1) | 1(5) | <0.001 |
| BMI, kg/m^2^, median (IQR) | 23 (22-26) | 22 (21-26) | 0.13 |
| BMI, n (%) |  |  | 0.67 |
| 0–24.9 kg/m^2^ | 35 (66) | 15 (75) |  |
| 25.0–39.9 kg/m^2^ | 16 (30.2) | 4 (20) |  |
| ≥40 kg/m^2^ | 0 (0) | 0 (0) |  |
| Unknown | 2 (7.5) | 1(5) |  |
| Data are reported as number (percentage), mean ± standard deviation or median (IQR, interquartile range) as appropriate  p Value: for the comparison between COVID-19 patient and healthy volunteers  *Heart disease* coronary artery disease, congestive heart failure, valvular disease, *Lung disease* asthma, COPD, requiring home O_2_ and any chronic lung condition, *Kidney disease* chronic kidney disease, baseline creatinine >1.5, *Immunocompromised condition* active cancer, chemotherapy, transplant and immunosuppressant agents, asplenic, *BMI* body mass index | | | |
