# Supplementary material for: Combination of WFDC2, CHI3L1, and KRT19 in Plasma Defines a Clinically Useful Molecular Phenotype Associated with Prognosis in Critically Ill COVID-19 Patients
Source: J Clin Immunol. 2022 Nov 4;43(2):286–98. doi: 10.1007/s10875-022-01386-3 (PMC9638294; doi:10.1007/s10875-022-01386-3)
Supplement: Supplementary file 11 — Supplementary file11 (DOCX 26.5 KB) [file 10875_2022_1386_MOESM11_ESM.docx]

| **Supplemental Table 3** Clinical and demographic characteristics of COVID-19 patients in 2nd discovery cohort | | | | |
| --- | --- | --- | --- | --- |
|  | Overall | Early recovery | Late recovery | p Value |
|  | (n=53) | (n=23) | (n=30) |  |
| Male sex, n (%) | 37 (69.8) | 16 (69.6) | 20 (66.7) | 0.82 |
| Age, median years (IQR) | 73 (62-78) | 69 (56-74) | 74 (67-80) | 0.02 |
| Age group, n (%) |  |  |  | 0.09 |
| 20–34 years | 0 (0) | 0 (0) | 0 (0) |  |
| 35–49 years | 3 (5.7) | 3 (13.4) | 0 (0) |  |
| 50–64 years | 13 (26.4) | 7 (30.4) | 7 (23.3) |  |
| 65–79 years | 28 (49.1) | 11 (47.8) | 15 (50) |  |
| Over 80 years | 10 (18.9) | 2 (8.7) | 8 (26.7) |  |
| Comorbidities, n (%) |  |  |  |  |
| Heart disease | 4 (7.7) | 1 (4.6) | 3 (10.3) | 0.42 |
| Lung disease | 10 (19.2) | 5 (21.7) | 5 (17.2) | 0.16 |
| Kidney disease | 8 (15.4) | 1 (4.6) | 7 (24.1) | 0.03 |
| Immunocompromised condition | 5 (9.6) | 1 (4.4) | 4 (13.7) | 0.25 |
| Hypertension | 24 (46.2) | 9 (37.5) | 15 (51.7) | 0.36 |
| Diabetes | 25 (48.1) | 8 (34.7) | 17 (58.6) | 0.08 |
| BMI, kg/m^2^, median (IQR) | 23 (22-26) | 24 (22-26) | 23 (22-26) | 0.25 |
| BMI, n (%) |  |  |  | 0.78 |
| 0–24.9 kg/m^2^ | 35 (66) | 14 (61.1) | 21 (70) |  |
| 25.0–39.9 kg/m^2^ | 16 (30.2) | 8 (34.7) | 8 (26.7) |  |
| ≥40 kg/m^2^ | 0 (0) | 0 (0) | 0 (0) |  |
| Unknown | 2 (7.5) | 1 (4.6) | 1 (3.3) |  |
| Laboratory data at inclusion |  |  |  |  |
| White blood cell (10^3^/L) | 8481 ± 568 | 7291 ± 902 | 8806 ± 671 | 0.07 |
| Platelet count (10^3^/L) | 178.8 ± 9.9 | 192.1 ± 16.2 | 171.2 ± 12.3 | 0.27 |
| D-dimer (μg/mL) | 10.8 ± 5.4 | 2.2 ± 0.5 | 16.1 ± 8.7 | 0.02 |
| Creatinine (mg/dL) | 1.2 ± 0.2 | 0.7 ± 0.2 | 1.5 ± 0.3 | 0.15 |
| Bilirubin (mg/dL) | 0.6 ± 0.1 | 0.5 ± 0.2 | 0.6 ± 0.1 | 0.06 |
| LDH (IU/L) | 441.3 ± 16.9 | 391.8 ± 18.2 | 469.9 ± 22.7 | 0.02 |
| CRP (mg/dL) | 7.9 ± 0.9 | 5.5 ± 0.9 | 8.9 ± 1.1 | 0.03 |
| Severity of illness |  |  |  |  |
| P/F ratio | 182.3 ± 11.7 | 226.5 ± 17.4 | 154.3 ± 11.2 | <0.001 |
| SOFA score, median (IQR) | 5 (3-6) | 3 (2-5) | 6 (4-7) | 0.006 |
| Acuity max score |  |  |  | <0.001 |
| 1=28-day mortality | 5 (9.6) | 0 (0) | 5 (16.7) |  |
| 2=Intubated/ventilated, survived | 44 (83) | 19 (82.6) | 25 (83.3) |  |
| 3=Hospitalized, O_2_ required, survived | 4 (7.5) | 4 (17.4) | 0 (0) |  |
| 4=Hospitalized, no O_2_ required, survived | 0 (0) | 0 (0) | 0 (0) |  |
| 5=Discharged/Not hospitalized, survived | 0 (0) | 0 (0) | 0 (0) |  |
| Steroid treatment before inclusion, n (%) | 33 (67.3) | 11 (47.8) | 22 (73.3) | 0.71 |
| Number of days since onset, (IQR) | 9 (8-11) | 10 (9-12) | 9 (8-11) | 0.19 |
| Outcome |  |  |  |  |
| 28-day mortality, n (%) | 5 (9.6) | 0 (0) | 5 (16.7) | <0.001 |
| Data are reported as number (percentage), mean ± standard deviation or median (IQR, interquartile range) as appropriate  p Value: for the comparison between early recovery and late recovery group  *Heart disease* coronary artery disease, congestive heart failure, valvular disease, *Lung disease* asthma, COPD, requiring home O_2_ and any chronic lung condition, *Kidney disease* chronic kidney disease, baseline creatinine >1.5, *Immunocompromised condition* active cancer, chemotherapy, transplant and immunosuppressant agents, asplenic, *BMI* body mass index, *LDH* lactate dehydrogenase, *CRP* C-reactive protein, *P/F* PaO_2_/FIO_2_, *SOFA* Sequential Organ Failure Assessment | | | | |
