# Supplementary material for: Combination of WFDC2, CHI3L1, and KRT19 in Plasma Defines a Clinically Useful Molecular Phenotype Associated with Prognosis in Critically Ill COVID-19 Patients
Source: J Clin Immunol. 2022 Nov 4;43(2):286–98. doi: 10.1007/s10875-022-01386-3 (PMC9638294; doi:10.1007/s10875-022-01386-3)
Supplement: Supplementary file 12 — Supplementary file12 (DOCX 21 KB) [file 10875_2022_1386_MOESM12_ESM.docx]

| **Supplemental Table 4** Clinical and demographic characteristics of COVID-19 patients in validation cohort | | | |
| --- | --- | --- | --- |
|  | COVID-19 patients | Healthy controls | p Value |
|  | (n=113) | (n=16) |  |
| Male sex, n (%) | 80 (70.8) | 12 (75) | 0.12 |
| Age, median years (IQR) | 65 (55-74) | 57 (54-70) | 0.14 |
| Age group, n (%) |  |  | 0.61 |
| 20–34 years | 1 (0.8) | 0 (0) |  |
| 35–49 years | 11 (9.7) | 3 (18.8) |  |
| 50–64 years | 44 (38.9) | 8 (50) |  |
| 65–79 years | 47 (41.6) | 4 (25) |  |
| Over 80 years | 10 (8.9) | 1 (6.3) |  |
| Comorbidities, n (%) |  |  |  |
| Heart disease | 12 (10.6) | 2 (12.5) | 0.68 |
| Lung disease | 12 (10.6) | 0 (0) | 0.2 |
| Kidney disease | 12 (10.6) | 0 (0) | 0.2 |
| Immunocompromised condition | 4 (3.5) | 0 (0) | 0.47 |
| Hypertension | 47 (41.6) | 4 (25) | 0.34 |
| Diabetes | 41 (36.3) | 1 (6.3) | 0.02 |
| BMI, kg/m^2^, median (IQR) | 25 (22-28) | 22 (20-25) | 0.02 |
| BMI, n (%) |  |  | 0.09 |
| 0–24.9 kg/m^2^ | 49 (43.4) | 12 (75) |  |
| 25.0–39.9 kg/m^2^ | 58 (51.3) | 3 (18.8) |  |
| ≥40 kg/m^2^ | 1 (0.8) | 0 (0) |  |
| Unknown | 5 (4.4) | 1 (6.3) |  |
| Data are reported as number (percentage), mean ± standard deviation or median (IQR, interquartile range) as appropriate  p Value: for the comparison between COVID-19 patient and healthy control  *Heart disease* coronary artery disease, congestive heart failure, valvular disease, *Lung disease* asthma, COPD, requiring home O_2_ and any chronic lung condition, *Kidney disease* chronic kidney disease, baseline creatinine >1.5, *Immunocompromised condition* active cancer, chemotherapy, transplant and immunosuppressant agents, asplenic, *BMI* body mass index | | | |
