# Supplementary material for: Combination of WFDC2, CHI3L1, and KRT19 in Plasma Defines a Clinically Useful Molecular Phenotype Associated with Prognosis in Critically Ill COVID-19 Patients
Source: J Clin Immunol. 2022 Nov 4;43(2):286–98. doi: 10.1007/s10875-022-01386-3 (PMC9638294; doi:10.1007/s10875-022-01386-3)
Supplement: Supplementary file 13 — Supplementary file13 (DOCX 25.2 KB) [file 10875_2022_1386_MOESM13_ESM.docx]

| **Supplemental Table 5** Clinical and demographic characteristics of COVID-19 patients in validation cohort | | | | |
| --- | --- | --- | --- | --- |
|  | Overall | Early recovery | Late recovery | p Value |
|  | (n=113) | (n=66) | (n=47) |  |
| Male sex, n (%) | 80 (70.8) | 48 (72.7) | 32 (68.1) | 0.41 |
| Age, years, median (IQR) | 65 (55-74) | 61 (54-71) | 69 (58-78) | 0.001 |
| Age group, n (%) |  |  |  | 0.02 |
| 20–34 years | 1 (0.8) | 1 (1.5) | 0 (0) |  |
| 35–49 years | 11 (9.7) | 8 (12.1) | 3 (9.7) |  |
| 50–64 years | 44 (38.9) | 30 (45.5) | 14 (29.8) |  |
| 65–79 years | 47 (41.6) | 24 (36.4) | 23 (48.9) |  |
| Over 80 years | 10 (8.9) | 3 (4.6) | 7 (14.9) |  |
| Comorbidities, n (%) |  |  |  |  |
| Heart disease | 12 (10.6) | 7 (10.6) | 5 (10.6) | 0.98 |
| Lung disease | 12 (10.6) | 7 (10.6) | 5 (10.6) | 0.75 |
| Kidney disease | 12 (10.6) | 6 (9.1) | 6 (12.8) | 0.54 |
| Immunocompromised condition | 4 (3.5) | 2 (3) | 2 (4.3) | 0.75 |
| Hypertension | 47 (41.6) | 25 (37.9) | 22 (46.8) | 0.24 |
| Diabetes | 41 (36.3) | 24 (36.4) | 17 (36.2) | 0.87 |
| BMI, kg/m^2^, median (IQR) | 25 (22-28) | 25 (22-27) | 25 (22-28) | 0.86 |
| BMI, n (%) |  |  |  | 0.16 |
| 0–24.9 kg/m^2^ | 49 (43.4) | 28 (42.4) | 21 (44.7) |  |
| 25.0–39.9 kg/m^2^ | 58 (51.3) | 33 (50) | 25 (53.2) |  |
| ≥40 kg/m^2^ | 1 (0.8) | 0 (0) | 1 (2.1) |  |
| Unknown | 5 (4.4) | 5 (7.6) | 0 (0) |  |
| Laboratory data at inclusion |  |  |  |  |
| White blood cell (10^3^/L) | 9133 ± 516 | 8020 ± 412 | 10696 ± 1064 | 0.22 |
| Platelet count (10^3^/L) | 86.3 ± 8.7 | 94.9 ± 12.3 | 74.1 ± 11.8 | 0.04 |
| D-dimer (μg/mL) | 10.3 ± 3.3 | 6.6 ± 3.7 | 15.6 ± 6.1 | 0.02 |
| Creatinine (mg/dL) | 1.0 ± 0.1 | 0.8 ± 0.1 | 1.3 ± 0.2 | 0.03 |
| Bilirubin (mg/dL) | 0.6 ± 0.1 | 0.6 ± 0.1 | 0.7 ± 0.1 | 0.58 |
| LDH (IU/L) | 475.7 ± 19.5 | 434.1 ± 23.7 | 534.2 ± 31.4 | 0.02 |
| CRP (mg/dL) | 9.2 ± 0.7 | 8.9 ± 0.8 | 9.8 ± 1.0 | 0.36 |
| Severity of illness |  |  |  |  |
| P/F ratio | 186.7 ± 7.6 | 215.2 ± 10.8 | 146.6 ± 6.9 | <0.001 |
| SOFA score, median (IQR) | 5 (3-6) | 3 (4-5) | 6 (4-7) | 0.22 |
| Acuity max score |  |  |  | <0.001 |
| 1=28-day mortality | 16 (14.2) | 16 (24.2) | 0 (0) |  |
| 2=Intubated/ventilated, survived | 97 (85.8) | 50 (75.8) | 47 (100) |  |
| 3=Hospitalized, O_2_ required, survived | 0 (0) | 0 (0) | 0 (0) |  |
| 4=Hospitalized, no O_2_ required, survived | 0 (0) | 0 (0) | 0 (0) |  |
| 5=Discharged/Not hospitalized, survived | 0 (0) | 0 (0) | 0 (0) |  |
| Steroid treatment before inclusion, n (%) | 76 (67.2) | 47 (71.2) | 29 (61.7) | 0.06 |
| Number of days since onset, (IQR) | 9 (7-11) | 9 (8-11) | 8 (7-10) | 0.83 |
| Outcome |  |  |  |  |
| 28-day mortality, n (%) | 12 (10.7) | 0 (0) | 16 (24.2) | <0.001 |
| Data are reported as number (percentage), mean ± standard deviation or median (IQR, interquartile range) as appropriate p Value: for the comparison between early recovery and late recovery group  *Heart disease* coronary artery disease, congestive heart failure, valvular disease, *Lung disease* asthma, COPD, requiring home O_2_ and any chronic lung condition, *Kidney disease* chronic kidney disease, baseline creatinine >1.5, *Immunocompromised condition* active cancer, chemotherapy, transplant and immunosuppressant agents, asplenic, *BMI* body mass index, *LDH* lactate dehydrogenase, *CRP* C-reactive protein, *P/F* PaO_2_/FIO_2_, *SOFA* Sequential Organ Failure Assessment | | | | |
