# Supplementary material for: Combination of WFDC2, CHI3L1, and KRT19 in Plasma Defines a Clinically Useful Molecular Phenotype Associated with Prognosis in Critically Ill COVID-19 Patients
Source: J Clin Immunol. 2022 Nov 4;43(2):286–98. doi: 10.1007/s10875-022-01386-3 (PMC9638294; doi:10.1007/s10875-022-01386-3)
Supplement: Supplementary file 14 — Supplementary file14 (DOCX 21 KB) [file 10875_2022_1386_MOESM14_ESM.docx]

| **Supplemental Table 6** Clinical and demographic characteristics of COVID-19 patients treated without invasive mechanical ventilation | |
| --- | --- |
|  | COVID-19 patients |
|  | (n=9) |
| Male sex, n (%) | 6 (66.7) |
| Age, median years (IQR) | 71 (55.5-79) |
| Age group, n (%) |  |
| 20–34 years | 0 (0) |
| 35–49 years | 1 (11.1) |
| 50–64 years | 2 (22.2) |
| 65–79 years | 5 (55.6) |
| Over 80 years | 1 (11.1) |
| Comorbidities, n (%) |  |
| Heart disease | 1 (11.1) |
| Lung disease | 1 (11.1) |
| Kidney disease | 0 (0) |
| Immunocompromised condition | 0 (0) |
| Hypertension | 4 (44.4) |
| Diabetes | 3 (33.3) |
| BMI, kg/m^2^, median (IQR) | 26 (23-32) |
| BMI, n (%) |  |
| 0–24.9 kg/m^2^ | 2 (22.2) |
| 25.0–39.9 kg/m^2^ | 7 (77.8) |
| ≥40 kg/m^2^ | 0 (0) |
| Unknown | 0 (0) |
| Data are reported as number (percentage), mean ± standard deviation or median (IQR, interquartile range) as appropriate  *Heart disease* coronary artery disease, congestive heart failure, valvular disease, *Lung disease* asthma, COPD, requiring home O_2_ and any chronic lung condition, *Kidney disease* chronic kidney disease, baseline creatinine >1.5, *Immunocompromised condition* active cancer, chemotherapy, transplant and immunosuppressant agents, asplenic, *BMI* body mass index | |
