# Supplementary material for: Combination of WFDC2, CHI3L1, and KRT19 in Plasma Defines a Clinically Useful Molecular Phenotype Associated with Prognosis in Critically Ill COVID-19 Patients
Source: J Clin Immunol. 2022 Nov 4;43(2):286–98. doi: 10.1007/s10875-022-01386-3 (PMC9638294; doi:10.1007/s10875-022-01386-3)
Supplement: Supplementary file 15 — Supplementary file15 (DOCX 22 KB) [file 10875_2022_1386_MOESM15_ESM.docx]

| **Supplemental Table 7** Patients characteristics by latent class analysis | | | | |
| --- | --- | --- | --- | --- |
|  | α-phenotype  (n=60) | β-phenotype  (n=45) | γ-phenotype  (n=8) | p-Value^1^ |
| Age, years, median (IQR) | 61 (53, 69) | 72 (61, 78) | 59 (57, 71) | <0.05* |
| Sex, male, n (%) | 43 (72) | 33 (73) | 4 (50) | 0.44 |
| BMI, kg/m^2^, median (IQR) | 25.7 (22.9, 28.2) | 25.0 (22.8, 28.3) | 23.9 (21.4, 25.3) | 0.34 |
| Four key proteins |  |  |  |  |
| WFDC2 (ng/mL) | 9.1 (7.1, 11.7) | 20.1 (12.3, 28.0) | 211.6 (154,7, 266.8) | <0.05* |
| CHI3L1 (ng/mL) | 273.7 (16.7, 437.1) | 910.6 (716.6, 1195.9) | 1613.6 (1220.3, 1986.4) | <0.05* |
| GDF15 (pg/mL) | 1691 (1342, 2765) | 3946 (2838, 5310) | 15286 (9973, 22467) | <0.05* |
| KRT19 (pg/mL) | 1638 (1052, 2472) | 3768 (2974, 5742) | 11697 (8464, 13349) | <0.05* |
| Laboratory data |  |  |  |  |
| WBC (×100/μL) | 56 (7, 90) | 46 (9, 94) | 62 (36, 119) | 0.70 |
| PLT (×1000/μL) | 29 (21, 144) | 27 (21, 184) | 18 (13, 101) | 0.15 |
| CRP (mg/dL) | 7 (3, 12) | 9 (5, 15) | 8 (3, 15) | 0.10 |
| Cre (mg/dL) | 0.62 (0.54, 0.77) | 0.82 (0.69, 1.14) | 3.34 (2.10, 4.48) | <0.05* |
| T-Bilirubin (mg/dL) | 0.58 (0.40, 0.79) | 0.60 (0.40, 0.70) | 0.54 (0.38, 1.41) | 0.97 |
| D-dimer (μg/dL) | 1 (1, 2) | 2 (1, 8) | 22 (3, 51) | <0.05* |
| Fibrinogen (mg/dL) | 552 (468, 629) | 578 (451, 652) | 291 (192, 534) | 0.09 |
| PT-INR | 1.08 (1.00, 1.19) | 1.12 (1.03, 1.22) | 1.44 (1.16, 1.84) | 0.046* |
| Comorbidities, n (%) |  |  |  |  |
| Diabetes | 17 (28) | 21 (47) | 3 (38) | 0.14 |
| Hypertension | 22 (37) | 21 (47) | 4 (50) | 0.50 |
| Hyperlipidemia | 21 (35) | 8 (18) | 1 (12) | 0.11 |
| Heart disease | 5 (8.3) | 5 (11) | 2 (25) | 0.29 |
| Lung disease | 6 (10) | 5 (11) | 1 (12) | >0.99 |
| Renal disease | 2 (3.3) | 5 (11) | 5 (62) | <0.05* |
| ECMO, n (%) | 1 (1.7) | 4 (8.9) | 1 (12) | 0.13 |
| Tracheostomy, n (%) | 11 (18) | 13 (29) | 3 (38) | 0.27 |
| Hospital days, median (IQR) | 9 (6, 12) | 12 (9, 17) | 12 (5, 20) | 0.02* |
| Mortality, n (%) |  |  |  |  |
| 28-day mortality | 0 (0) | 8 (18) | 4 (50) | <0.05* |
| Hospital mortality | 2 (3.3) | 10 (22) | 4 (50) | <0.05* |
| Data are presented as the median (25th-75th percentile) or as number (%)  ^1^Significance was determined by Kruskal-Wallis rank sum test or Fisher’s exact test  *IQR* interquartile range, *BMI* body mass index, *WBC* white blood cell, *PLT* platelets, *CRP* C-reactive protein, *Cre* creatinine, *T* total, *PT-INR* prothrombin time-international normalized ratio, *ECMO* extracorporeal membrane oxygenation  *Statistically significant (p<0.05) | | | | |
